# Appraisal Tools for Clinical Practice Guidelines: A Systematic Review

**DOI:** 10.1371/journal.pone.0082915

**Published:** 2013-12-09

**Authors:** Ulrich Siering, Michaela Eikermann, Elke Hausner, Wiebke Hoffmann-Eßer, Edmund A. Neugebauer

**Affiliations:** 1 Institute for Quality and Efficiency in Health Care (IQWiG), Cologne, Germany; 2 Institute for Research in Operative Medicine (IFOM), Faculty of Health, Department of Medicine, Witten/Herdecke University, Cologne, Germany; National Taiwan University, Taiwan

## Abstract

**Introduction:**

Clinical practice guidelines can improve healthcare processes and patient outcomes, but are often of low quality. Guideline appraisal tools aim to help potential guideline users in assessing guideline quality. We conducted a systematic review of publications describing guideline appraisal tools in order to identify and compare existing tools.

**Methods:**

Among others we searched MEDLINE, EMBASE and the Cochrane Database of Systematic Reviews from 1995 to May 2011 for relevant primary and secondary publications. We also handsearched the reference lists of relevant publications.

On the basis of the available literature we firstly generated 34 items to be used in the comparison of appraisal tools and grouped them into thirteen quality dimensions. We then extracted formal characteristics as well as questions and statements of the appraisal tools and assigned them to the items.

**Results:**

We identified 40 different appraisal tools. They covered between three and thirteen of the thirteen possible quality dimensions and between three and 29 of the possible 34 items. The main focus of the appraisal tools were the quality dimensions “evaluation of evidence” (mentioned in 35 tools; 88%), “presentation of guideline content” (34 tools; 85%), “transferability” (33 tools; 83%), “independence” (32 tools; 80%), “scope” (30 tools; 75%), and “information retrieval” (29 tools; 73%). The quality dimensions “consideration of different perspectives” and “dissemination, implementation and evaluation of the guideline” were covered by only twenty (50%) and eighteen tools (45%) respectively.

**Conclusions:**

Most guideline appraisal tools assess whether the literature search and the evaluation, synthesis and presentation of the evidence in guidelines follow the principles of evidence-based medicine. Although conflicts of interest and norms and values of guideline developers, as well as patient involvement, affect the trustworthiness of guidelines, they are currently insufficiently considered. Greater focus should be placed on these issues in the further development of guideline appraisal tools.

## Introduction

Clinical practice guidelines (hereafter referred to as “guidelines”) are defined by the Institute of Medicine as “statements that include recommendations intended to optimize patient care that are informed by a systematic review of evidence and an assessment of the benefits and harms of alternative care options” [1]. Beyond that, guidelines are used for a variety of purposes, for example, as a means to measure and improve the quality of care, to resolve malpractice claims, to contribute to the development of clinical decision aids or to support policy makers in the allocation of healthcare resources [1].

There is evidence to suggest that, when rigorously developed, guidelines have the power to translate the complexity of scientific research findings and other evidence into recommendations for healthcare action [2-5]. 

Several studies have shown that guidelines can improve healthcare processes and patient outcomes. Grimshaw, Eccles, and Tetroe 2004 conducted a systematic review of the effectiveness and costs of various guideline development, dissemination and implementation strategies. The majority (86.6%) of the 235 studies included in their review reported improvements in health care [6,7]. Two other systematic reviews reported similar results [8,9]. However, all of the authors noted that the studies included were of low methodological quality. 

The AGREE Collaboration defines guideline quality as “the confidence that the potential biases of guideline development have been addressed adequately and that the recommendations are both internally and externally valid, and are feasible for practice” [10]. This definition has been widely adopted in the scientific literature [11,12].

Studies investigating the methodological quality of guidelines have often reported low quality and no, or only modest, improvement in quality over time [13-17].

Potential deficits of guidelines include:

- conflicting recommendations [18-26],
- insufficient consideration of relevant patient characteristics (e.g., multimorbidity or ethnic differences) [27-30],
- low quality of the evidence underlying the recommendations [31-35],
- lack of transparency of methods applied by guideline developers, especially concerning the derivation of recommendations and the determination of their strength [1],
- inadequate management of potential conflicts of interest [36-41].

Several groups, such as the Guidelines International Network [42], the Institute of Medicine [1], the World Health Organization [43], the National Institute for Health and Clinical Excellence [44], the Scottish Intercollegiate Guidelines Network [45], many medical societies [46-51], as well as individual experts in the field [12,52-55], have proposed manuals defining standards for guideline developers in order to increase guideline quality. Overall, these manuals address the following key elements in the development process: establishment of a multidisciplinary guideline development group, consumer involvement, identification of clinical questions or problems, conduct of systematic searches and appraisal of the evidence retrieved, procedures for drafting recommendations, external consultation, and ongoing reviewing and updating [56].

Parallel to the production of manuals for the development of high-quality guidelines, tools for their appraisal have been developed. These tools aim to help potential guideline users to assess guideline quality. The AGREE II Instrument – the guideline appraisal tool used most often internationally – contains questions covering the areas (1) scope and purpose, (2) stakeholder involvement, (3) rigour of development, (4) clarity of presentation, (5) applicability, and (6) editorial independence [57].

Graham 2000 identified and compared guideline appraisal tools in a systematic review [58], which was updated by Vlayen in 2005 [59]. Vlayen identified 24 different tools containing questions that could be grouped into ten quality dimensions with 50 different items. Four of the 24 tools covered all of the guideline dimensions, but only four were validated and none assessed the evidence base of the clinical content of the guidelines. The authors stated that “the results of the search for evidence, the correct use of inclusion and exclusion criteria, and the critical appraisal of the retrieved evidence are not validated. Therefore, a major conclusion of this review is that in order to evaluate the quality of the clinical content and more specifically the evidence base of a clinical practice guideline, verification of the completeness and the quality of the literature search and its analysis has to be added to the process of validation by an appraisal instrument.” 

The aims of this systematic review were to identify and compare existing guideline appraisal tools to see if the landscape of tools had changed. This comparison can then be used to support decision-making by clinicians, patients and policy makers concerning the selection of the most appropriate tool, as well as to identify potential for improvement.

## Methods

We searched for relevant primary and secondary publications (systematic and narrative reviews) in MEDLINE, EMBASE, the Cochrane Database of Systematic Reviews (Cochrane Reviews), the Database of Abstracts of Reviews of Effects (Other Reviews), the Health Technology Assessment Database (Technology Assessments), the NHS Economic Evaluation Database, and the Cochrane Methodology Register. The systematic search was limited to publications in German and English published after 1994. The search in all databases was performed in May 2011. The search strategy included, among others, the search terms “guideline”, “appraisal”, “guideline adherence”, “quality”, “evidence based” and “evaluation”. The full search strategy, which was developed by an information specialist (EH), is attached to this publication as online File S1. In addition, we scrutinized the reference lists of the relevant primary and secondary publications retrieved in the above search to identify further publications.

We included articles with the following characteristics:

- Publication described the most recent version of an appraisal tool for clinical guidelines
- Availability of a full-text document (e.g., journal article or internet file).

Articles were excluded that only described the content of guidelines, the guideline development process or the application of an appraisal tool already identified in another publication.

Two reviewers (US, WHE) independently screened titles and abstracts of the retrieved citations to identify potentially eligible primary and secondary publications. The full texts were obtained and independently evaluated by the same two reviewers. Disagreements were resolved by consensus.

Since the primary aim of this review was to identify existing guideline appraisal tools and to describe and compare their formal and content characteristics, no risk of bias assessment was conducted for the publications included.

The content analysis was a two-stage process. The first stage involved the generation of items to be used in the comparison of appraisal tools by compilation of a list of all questions and statements from each of the tools included. These were grouped into common questions and statements and assigned to an item label. The items were then assigned to broader common categories, named quality dimensions, which were largely derived from Cluzeau et al. 1999 [60], Graham 2000 [58] and Vlayen 2005 [59]. 

The individual steps of the content analysis procedures were always conducted by one person (US) and checked by another (WHE). Disagreements were resolved by consensus.

We identified 34 individual items and assigned them to thirteen quality dimensions (see Table 1 for detailed definitions).

**Table 1:** Quality dimensions and items for guideline appraisal.

**Quality dimensions** / Item label	**Definition**
**1. Information retrieval**	
Health questions and outcomes	Description of clinical health questions and relevant outcomes of the guideline
Literature search	Search for literature and other evidence
Literature selection	Criteria used to include and exclude literature and other evidence
**2. Evaluation of evidence**	
Grading of evidence	Grading of the evidence, which may or may not include a statement about the strength of evidence (LoE)
Consistency between evidence and recommendations	Studies results are reported correctly in the guideline and support the recommendations
**3. Consideration of different perspectives**	
Norms and values	Discussion of influence of norms and values on guideline development
Expert knowledge	Evaluation of expert opinion and clinical experience
Patient perspectives	Consideration of views and preferences of the target population in the guideline development process
**4. Formulation of recommendations**	
Formulation of recommendations	Methods used in formulating recommendations which may or may not include a statement about the strength of recommendations (GoR)
**5. Transferability**	
Comparability	Patients, interventions and settings in the studies were comparable to those targeted by the recommendations
Costs	Consideration of resource implications of applying the recommendations
Barriers and facilitators	Description of barriers and facilitators to guideline application (compatibility of guideline with local norms and values; professional’s training, skill, and experience; availability of drugs or technology; local adaptation or modification of the guideline)
**6. Presentation of guideline content**	
Benefits and harms	Presentation of health benefits, side effects, and harms of the recommended action
Link to evidence	Explicit link between the recommendations and the supporting evidence
**7. Alternatives**	
Options for management	Presentation of alternative options for management of the condition or health issues
Exceptions	Description of situations in which guidelines may not apply
Patient preferences	Consideration of patient preferences in the application of guideline recommendations
**8. Reliability**	
Independent Review	External peer review before publication
Pilot test	Pilot test of the guideline prior to release
**9. Scope**	
Rationale and objective	Description of the rationale or reason for guideline development and description of the goal or objective of the guideline
Guideline topic	Topic, or health problem, or technology dealt with
Practice setting	Practice setting for which the guideline is intended
Patient population	Patient population for whom the guideline is intended
Provider population	Group of health care providers for whom the guideline is intended
**10. Independence**	
Guideline development group	Individuals and/or disciplines, or occupations represented in the guideline development group and their function in the group
Guideline development organization and funding	Organization or group who developed the guideline and sources of funding
Conflicts of interest	Consideration of (potential) conflicts of interest related to the individuals developing the guideline
**11. Clarity and presentation**	
Clarity	Clear wording of the guideline and the recommendations
Presentation	Easily identifiable recommendations (e. g., summarized in a box, bold text, underlined). Graphical description of the stages and decisions in clinical care (clinical algorithm).
**12. Updating**	
Currentness	Currentness of the evidence of the guideline Date of issue of guideline and or date guideline becomes invalid
Scheduled review	Procedure for updating the guideline
**13. Dissemination, Implementation, Evaluation**	
Dissemination	Distribution of the guideline to intended users
Implementation	Strategies to implement the guideline
Evaluation	Evaluation of the guideline and the adherence to the guideline once it has been implemented

For the second stage of the analysis, we (US, WHE) extracted the following information from each publication:

(1) Formal characteristics of the appraisal tool.

These included language, the use of existing appraisal tools for tool development, number of items and domains, possible answers, number of appraisers, calculation of domain scores and overall assessment, information on the development and validation of the appraisal tool, as well as publication in a journal.

(2) Questions and statements of the appraisal tools.

One reviewer (US) then assigned the questions and statements to the items identified during the first stage of the content analysis. A second reviewer (WHE) confirmed this step by once again checking the questions of each appraisal tool and the items to which they had been assigned. Disagreements were resolved by consensus. The numbers of quality dimensions and items covered by each appraisal tool were then compared.

The review was not registered in advance, nor has a review protocol been published.

## Results

### Selection of publications

**Figure 1:**
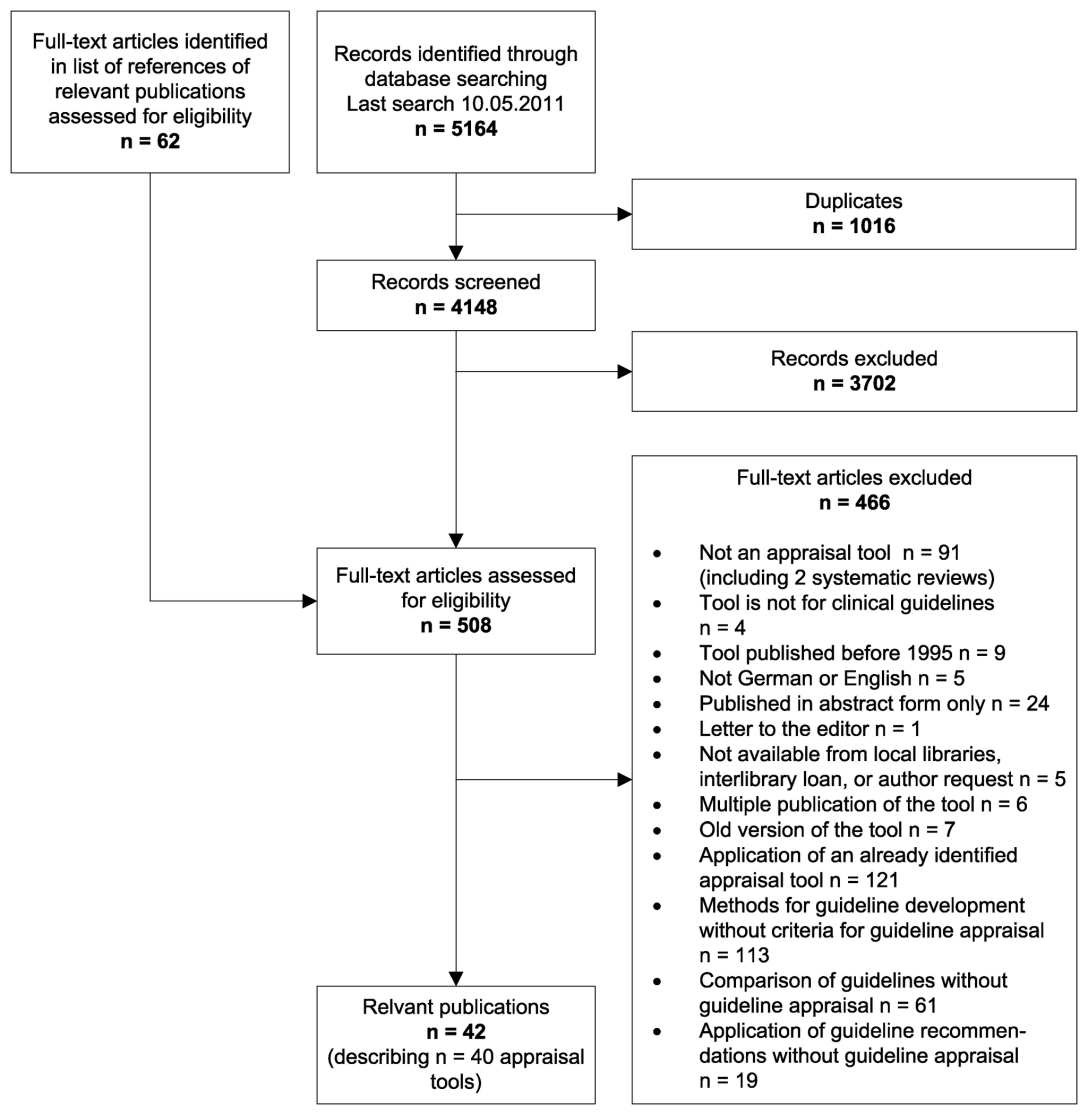
Flow chart for selection of appraisal tools.

We retrieved 5164 references from bibliographic databases and screened 446 full texts. In addition, we retrieved 62 further publications from the reference lists of the relevant primary and secondary publications. We identified a total of 42 eligible publications describing 40 different guideline appraisal tools (Figure 1). Excluded publications are listed in online File S2. Relevant secondary publications are listed in online File S3.

### Description of Appraisal Tools

Table 2 shows the main formal characteristics of the 40 appraisal tools considered. 38 were published in English and two in German. 26 named at least one other publication that had influenced their developmentand ten named the AGREE Instrument [10]; other publications mentioned included those by Hayward 1995, Wilson 1995 and Field 1992 [61-63]. 

**Table 2:** Formal characteristics of guideline appraisal tools.

**Appraisal tool**	**Language**	**Based on**	**Additional information on development of appraisal tool**	**Generic appraisal tool^*a*^**	**Subject of assessment**	**Number of questions (Domains)**	**Explanation of questions**	**Rating scale / Multiple choice / Additional comments**	**Number of appraiser**	**Domain scores / Overall assessment**	**Validation**	**Publication in journal**
ADAPTE 2009 [64]	EN	n. s.	yes	yes	GL / Rec.	43 (3)	SE	no / yes / no	n. s.	no / no	yes	no
AGREE II 2009 [57]	EN	[10]	yes	yes	GL	23 (6)	SE	yes / no / yes	2, better 4	yes / yes	yes	no
APWCA 2010 [81]	EN	n. s.	no	yes	GL	11 (-)	no	no / no / no	n. s.	no / no	no	yes
APA 2002[97]	EN	n. s.	no	yes	GL	47 (21)	SE	no / no / no	n. s.	no / no	no	yes
Baxter 2003 [82]	EN	[61,91,113]	no	yes	GL	12 (4)	SE	no / no / no	n. s.	no / no	no	yes
BÄK 1997 [83]	GE	n. s.	no	yes	GL	12 (-)	SE	no / no / no	n. s.	no / no	no	yes
Calder 1997 [107]	EN	[60-62,116,117]	no	yes	GL	26 (5)	no	no / yes / no	2	no / no	no	yes
Chong 2009 [66]	EN	[10,13]	no	yes	GL	11 (2)	no	yes**^*b*^** / yes**^*c*^** / no	2	no / no	yes	yes
Chou 2008[84,85]	EN	[10,14,118]	no	yes	GL	26 (5)	SE	no / no / no	n. s.	no / no	no	yes
Cluzeau 1999 [60]	EN	[63]	no	yes	GL	37 (3)	no	no / yes / no	n. s.	yes / yes	yes	yes
Cook 1998 [119]	EN	[61,62]	no	yes	GL	9 (3)	SE	no / no / no	n. s.	no / no	no	yes
DELBI 2008 [65]	GE	[10]	yes	yes	GL	34 (8)	CE	yes / no / no	2, better 4	yes / no	no	yes
Fields 2000 [86]	EN	[61,62]	no	yes	GL	8 (-)	no	no / no / no	n. s.	no / no	no	yes
Foy 2002 [87]	EN	[120,121]	yes	yes	Rec.	13 (-)	no	yes / no / no	n. s.	no / no	no	yes
Fretheim 2002 [98]	EN	[10,122]	no	yes	GL	8 (-)	CE	no / no / no	2	no / no	no	yes
GLIA 2011 [67]	EN	[10,13,60,63,120,121,123,124]	yes	yes	GL / Rec.	30 (9)	no	no / yes / yes	2	no / no	yes	no
Grilli 2000 [14]	EN	n. s.	no	yes	GL	3 (-)	CE	no / yes / no	2	no / no	yes	yes
Guyatt 2002 [88]	EN	n. s.	no	yes	GL	4 (-)	SE	no / no / no	n. s.	no / no	no	yes
Hargrove 2008 [108]	EN	[10,88,125-128]	no	yes	GL	18 (3)	SE	no / yes / yes	3	no / no	yes	yes
Hart 2002 [99]	EN	[10,13,14]	no	yes	GL	9 (-)	no	yes / no / no	n. s.	no / no	no	yes
Hasenfeld 2003 [100]	EN	[13]	no	yes	GL	30 (-)	no	no / yes / no	2	no / no	no	yes
Hayward 1995 [61,62]	EN	[129,130]	no	yes	Rec.	10 (3)	SE	no / no / no	n. s.	no / no	no	yes
Hindley 2005 [101]	EN	[10]	yes	yes	GL	18 (12)	no	yes / no / no	at least 2	no / yes	yes	yes
Kulig 2003 [102]	EN	[131]	yes	yes	GL	13 (3)	no	yes / no / no	2	yes / yes	yes	yes
Liddle 1996 [89]	EN	k. A	yes	yes	GL / Rec.	14 (3)	SE for 1 question	for some questions / for 1 question / yes	n. s.	no / no	yes	no
Linskey 2010 [90]	EN	n. s.	no	yes	GL	9 (-)	no	no / no / no	n. s.	no / no	no	yes
Marshall 2000 [91]	EN	[129,132,133]	no	yes	GL	9 (-)	SE	no / no / no	n. s.	no / no	no	yes
Mottur-Pilson 1995 [134]	EN	[63]	yes	yes	GL	18 (-)	no	no / yes / yes	n. s.	no / yes	no	yes
Nonino 2004 [92]	EN	[10,14]	no	yes	GL	6 (-)	no	no / no / no	n. s.	no / no	no	yes
Pentheroudakis 2008 [103]	EN	n. s.	no	(no)**^*d*^**	GL	24 (4)	SE	no / no / no	n. s.	no / no	no	yes
Sanderlin 2007 [93]	EN	n. s.	no	yes	GL	5 (-)	SE	no / no / no	n. s.	no / no	no	yes
Sanders 2000 [104]	EN	[135]	no	yes	GL	15 (3)	no	yes / no / no	n. s.	yes / yes	no	yes
Savoie 2000 [105]	EN	[113,136]	no	(no)**^*d*^**	GL	51 (15)	no	no / yes / yes	2	no / no	no	yes
Shaneyfelt 1999 [13]	EN	[116]	yes	yes	GL	25 (3)	no	no / yes / no	2	no / no	yes	yes
Shiffman 2003 [2]	EN	[63,131,137]	yes	yes	GL / Rec.	18 (-)	no	no / no / no	n. s.	no / no	no	yes
Veale 1999 [94]	EN	n. s.	no	yes	GL	7 (-)	no	no / no/ no	n. s.	no / no	no	yes
Ward 1996 [106]	EN	[63]	no	yes	GL	18 (8)	no	no / yes / no	n. s.	no / no	no	yes
Warriner 2011 [95]	EN	n. s.	no	yes	GL	11 (9)	SE	no / no/ no	n. s.	no / no	no	yes
WHO 2003 [43]	EN	n. s.	no	yes	GL	25 (8)	no	no / no / no	n. s.	no / no	no	no
Woolf 1995 [96]	EN	n. s.	no	yes	GL	10 (-)	no	no / no/ no	n. s.	no / no	no	yes

n. s. not specified; EN English; GE German; GL Guideline; Rec. Recommendation; SE: Some explanations; CE: Concrete explanations

a: A generic appraisal tool is a tool that can be used to appraise all kinds of clinical practice guidelines.b: For 4 of the 11 questions.c: For 7 of the 11 questions.d: The appraisal tool includes some disease-specific questions.

Eleven appraisal tools provided additional information on their development process. The number of questions in the tools ranged from three to 51. 23 tools grouped their questions into domains. The number of domains ranged from two to 21. Eighteen tools contained at least some explanation of their questions. 

Twenty tools used no specified scoring system, and twelve used a multiple choice answer, mostly a “yes/no” score, with or without the options ‘not sure’ or ‘not applicable’. Nine tools applied some form of scaling system. Six tools explicitly requested additional comments from guideline appraisers.

Thirteen appraisal tools recommended that guidelines should be appraised independently by at least two reviewers. 

The calculation of a quality score for the domains of an appraisal tool and a qualitative or quantitative overall assessment of the guideline were suggested by five and six tools respectively. Only eleven tools had been subject to any sort of validation studies and only six of these [13,60,64-67] had been validated more thoroughly. All but five appraisal tools were published in peer-reviewed journals. 

Content analysis

**Figure 2:**
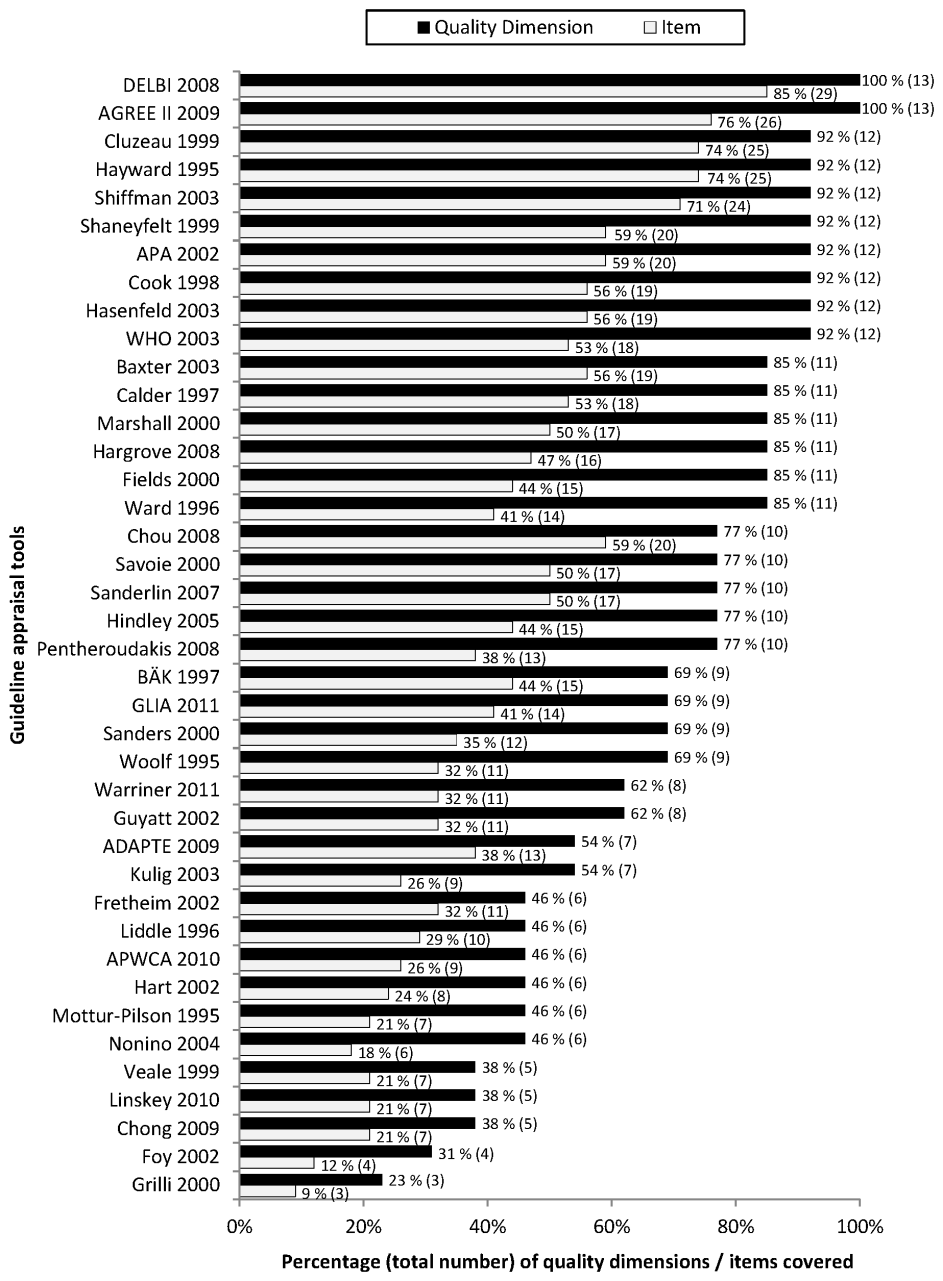
Percentage (total number) of quality dimensions / items covered by the guideline appraisal tools.

**Figure 3:**
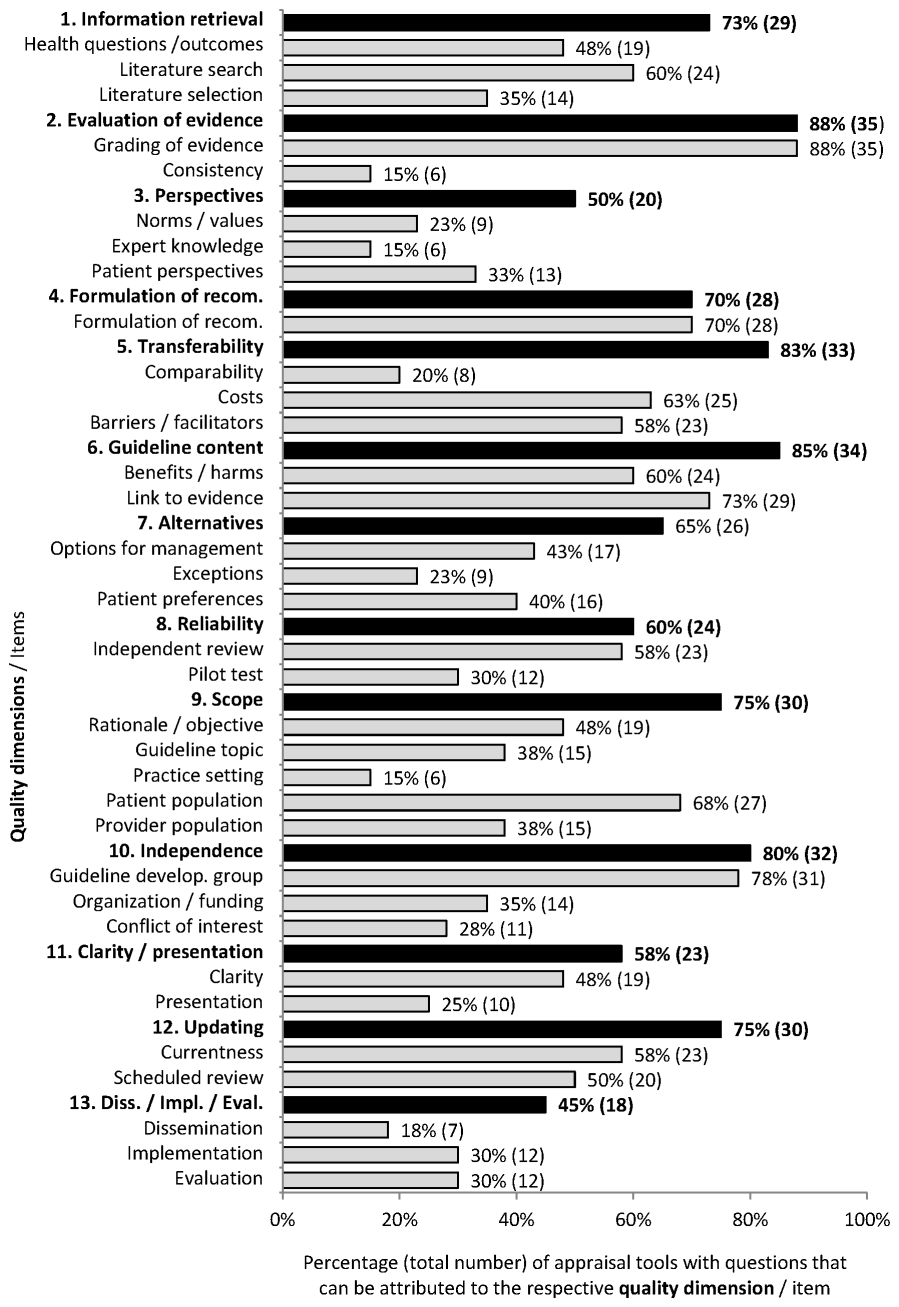
Percentage (total number) of appraisal tools with questions that can be attributed to the respective quality dimension / item.

Figures 2 and 3 compare the quality dimensions and items covered by the appraisal tools analysed. 

The tools varied considerably in terms of the number of quality dimensions covered. Ten (25%) covered at least twelve quality dimensions with at least one item; eleven (28%) covered only six or fewer quality dimensions.

The appraisal tools also differed in the extent to which each quality dimension was covered. Of the 34 possible items the number covered by each tool varied between three and 29 (Figure 2).

The quality dimensions “evaluation of evidence” (mentioned in 35 tools; 88%) and “information retrieval” (29 tools; 73%) were a main focus of the appraisal tools. However, the tools rarely assessed whether the study results were reported correctly in the guidelines and supported the recommendations (item “consistency” mentioned in six tools; 15%).

Another focus was the quality dimension “transferability” (33 tools; 83%) with the items “costs” (25 tools; 63%) and “barriers and facilitators” (23 tools; 58%). However, the tools rarely assessed whether patients, interventions and settings in the studies underlying the recommendations were comparable to those targeted by the recommendations (item “comparability” mentioned in eight tools; 20%).

Further quality dimensions covered by at least 70% of the appraisal tools were the dimensions “presentation of guideline content” (34 tools; 85%), “independence” (32 tools; 80%), “scope” (30 tools; 75%), “updating” (30 tools; 75%), and “formulation of recommendations” (28 tools; 70%). The item “composition of the guideline development group” in the quality dimension “independence” was covered frequently (32 tools; 80%), whereas few appraisal tools mentioned the item “consideration of (potential) conflicts of interest" related to the guideline development group (eleven tools; 28%).

The following two quality dimensions were covered by 50% or less of the appraisal tools: firstly, “consideration of different perspectives” (20 tools; 50%) with the items “patient perspectives” (thirteen tools; 33%), “norms and values” (nine tools; 23%), and “expert knowledge” (six tools; 15%), and secondly, “dissemination, implementation and evaluation of the guideline” (eighteen tools; 45%) (Figure 3).

A table with the complete content characteristics of the guideline appraisal tools is attached as online File S4.

## Discussion

### Main findings

The aim of this systematic review was to identify and compare existing guideline appraisal tools. We identified 40 different tools. Among those were 24 new tools not included in the systematic reviews by Graham 2000 [58] and Vlayen 2005 [59], as well as an additional three updated tools.

Most appraisal tools assess whether the literature search, the evaluation and synthesis of the evidence, and the reporting of the evidence in the guidelines are in accordance with the principles of evidence-based medicine. However, the guideline development process comprises more than the systematic compilation of the evidence on a relevant clinical question. Burgers et al 2002 stated that guideline development is a technical as well as social process [68]. The choice and interpretation of the evidence identified and the formulation of recommendations is affected by norms and values of the guideline development group [53,69-74]. Zuiderent-Jerak et al 2012 suggest that guidelines should reflect all knowledge, not just clinical trials [75]. However, few appraisal tools assess whether the formulation of recommendations is supported by a formal consensus process or whether the norms and values of the guideline development group are clearly stated.

Current standards for guideline development [1,42] point out that patients should be full members of the guideline development group. However, many of the appraisal tools fail to capture consumer involvement, i.e. do not assess whether patients’ views were considered in the guideline development group. 

Conflicts of interest may influence decisions in the health care system [76,77], also concerning the development of guidelines [36-38], and new and more stringent policies have been called for [42,55,78-80]. It is therefore surprising that only few appraisal tools assess whether conflicts of interest of members of the guideline development group have been recorded and addressed.

### Selection of an appraisal tool

Most of the appraisal tools included can be assigned to one of three groups:

1. Tools with general questions and with no or only a few appraisal criteria to decide whether the requirements of the questions are fulfilled [61,62,81-96].
2. Tools with specific questions or appraisal criteria to decide whether the requirements of the questions are fulfilled [2,13,14,43,65-67,97-106].
3. A small group of tools with specific questions and / or appraisal criteria with an additional qualitative appraisal [57,60,64,107,108]).

Differing results of guideline appraisals are more likely in cases where the questions of an appraisal tool are imprecise or specific criteria for answering the questions are lacking. This problem is particularly evident in the tools in the first group. For this reason the appraisal tools in the first group cannot be recommended for regular use.

It is also important to underline that appraisal tools in the first and second group mainly focus on methodological issues surrounding guideline development and reporting. However, they do not evaluate the quality of the clinical content itself [58,109]. For example, guideline appraisal tools in the first and second group assess whether the search strategy was reported in the guidelines, but they do not assess whether the search strategy was developed correctly or whether it was suited to identify evidence to answer the clinical question of the guideline. 

While rigorous development and explicit reporting of the guideline development process are necessary, they do not guarantee appropriate recommendations or better health outcomes for patients, as the methodological rigour and quality of the clinical content of a clinical practice guideline are not necessarily correlated [58,110-112]. 

Only the five tools of the third group are designed to solve this problem, at least to some degree. While their main focus is still the appraisal of methodological aspects of guideline development and reporting, they nevertheless require judgments on whether relevant quality aspects have been adequately implemented. For example, they assess not only whether the search strategy was reported but also require a qualitative statement on whether the strategy was appropriate [57,60,64,107,108], whether the evidence identified was appropriately summarized in the recommendations [60,64,107,108] or whether an appropriate formal process was used to arrive at the recommendations [57,60].

Appraisal tools differ in the number of items and quality dimensions covered. If the aim is to conduct a comprehensive guideline appraisal, the AGREE II tool [57] or the German-language DELBI tool [65] may represent the best choice. Both tools cover all thirteen quality dimensions. The AGREE II tool has also been thoroughly evaluated. 

However, an appraisal tool containing many quality dimensions may not necessarily represent the best choice in all cases. If the primary goal is to learn more about the applicability of a guideline, the GLIA tool [67] may be more suitable. This thoroughly evaluated tool appraises aspects that influence the applicability of a guideline. If the goal is to gain more information on the quality of the clinical content of a guideline, the ADAPTE tool [64] may be more suitable. This tool primarily includes questions that can be assigned to the quality dimensions “information retrieval” and “evaluation of evidence”. It has also been thoroughly evaluated, but demands considerable skill on the part of the guideline appraiser. Moreover, additional information not available in the guideline may be needed to answer the questions in this appraisal tool.

Depending on the problem being addressed, a tool containing only a few, but appropriate questions could be adequate. Furthermore, it may sometimes be advisable to omit some domains or items of an extensive appraisal tool. 

Information S4 provides details of the items and quality dimensions covered by the different appraisal tools. 

### Strengths and weaknesses of the review

Our review provides a comprehensive overview of guideline appraisal tools. It nevertheless has a number of limitations. 

A systematic search for appraisal tools is difficult, as there is no appropriate MESH or other term for appraisal tools. Because of the large number of appraisal tools used it is possible that not all appraisal tools were identified. Due to the comprehensive search strategy chosen, which included screening the reference lists of relevant primary and secondary publications, it is nevertheless unlikely that important and commonly used tools were not identified. 

The systematic search for appraisal tools was limited to tools published after 1994. In the late 1980s and early 1990s, the development of clinical practice guidelines became more common. With the definition of clinical practice guidelines by Field and Lohr in 1990 [113], a shared understanding of guidelines and guideline quality emerged that influenced the development of guidelines, as well as the development of appraisal tools. Authors of appraisal tools published before 1995 were probably not able to consider these developments.

We used the questions and statements contained in the appraisal tools, as well as the publications by Cluzeau 1999 [60], Graham 2000 [58] and Vlayen 2008 [59], to identify items and quality dimensions. According to this approach, the result of this review is a comparative description of the appraisal tools. There is no “gold standard” for the evaluation of appraisal tools. It is therefore possible that quality dimensions and items exist that were not identified, as they were not part of the publications and appraisal tools analysed, but may nevertheless be relevant for the appraisal of guideline quality. Furthermore, it was not always possible to clearly assign the questions or items of the appraisal tools to only one quality dimension. A further limitation of our review is that no external experts were consulted in the validation of the appraisal framework.

### Unanswered Questions and Future Research

The appraisal tools analysed cover several different aspects of guideline quality. All tools allow for the grading of guideline quality. However, it is uncertain whether all items and quality dimensions contribute equally to the quality of a guideline [58]. Further empirical studies are needed to answer the question as to which items and quality dimensions are essential for the assessment of guideline quality; for example, whether the external review of guidelines really improves their quality, whether conflicts of interest really lead to inappropriate recommendations or whether the explicit consideration of patient preferences really improves the patient-centeredness of a guideline.

In 2005 Vlayen stated “that in order to evaluate the quality of the clinical content and more specifically the evidence base of a clinical practice guideline, verification of the completeness and the quality of the literature search and its analysis has to be added to the process of validation by an appraisal instrument” [59]. Some appraisal tools have started to deal with this problem but have not solved it so far. 

The appraisal of the quality of the clinical content of guidelines is time-consuming, requires highly qualified personnel and may need additional information not available in the guidelines themselves. For example, an information specialist may be needed for appraisal of the appropriateness of a search strategy, it may be necessary to repeat a literature search to verify the completeness of the search results or the analysis of the literature identified has to be repeated to prove its correctness. 

Some working groups have started to deal with the appraisal of the clinical content of a guideline [114,115], but it remains unclear whether the assessment of the evidence base can be included in guideline appraisal tools in their current form. Further research will have to clarify whether and how overall appraisal of the clinical content of a guideline can be included in guideline appraisal tools with a reasonable use of resources.

## Conclusions

Appraisal tools differ in the number of items and quality dimensions covered and some tools cover some quality dimensions better than others. The most comprehensively validated appraisal tool is the AGREE II instrument, but the final choice of the appropriate tool depends on the research question. Nevertheless, appraisal tools containing unspecific questions and / or lacking criteria for answering the questions should not be applied. When choosing an appraisal tool it is important to keep in mind that their main focus is the appraisal of methodological aspects of guideline development and not the evaluation of the evidence base underlying a clinical practice guideline; further research should clarify whether and how an overall appraisal of the clinical content of a guideline can be performed.

Although conflicts of interest and norms and values of guideline developers, as well as patient involvement, affect the trustworthiness of guidelines, they are currently insufficiently assessed in guideline appraisal tools. They should thus be considered essential items in the further development of such tools.

## Supporting Information

Checklist S1 **PRISMA Checklist.** (PDF)

File S1 **Search strategy.**
(PDF)

File S2 **Excluded studies (ordered by reasons for exclusion).**
(PDF)

File S3 **Relevant secondary publications.**
(PDF)

File S4 **Content characteristics of guideline appraisal tools.**
(XLS)

## References

[1] Graham RM, Mancher M, Miller-Wolman D, Greenfield S, Steinberg E (2011) Clinical practice guidelines we can trust. Washington: National Academies Press. 24983061

[2] Shiffman RN, Shekelle P, Overhage JM, Slutsky J, Grimshaw J et al. (2003) Standardized reporting of clinical practice guidelines: a proposal from the Conference on Guideline Standardization. Ann Intern Med 139: 493-498. doi:10.7326/0003-4819-139-6-200309160-00013. PubMed: 13679327. 13679327

[3] Haines A, Jones R (1994) Implementing findings of research. BMJ 308: 1488-1492. doi:10.1136/bmj.308.6942.1488. PubMed: 8019284. 8019284 PMC2540317

[4] Shekelle PG, Kravitz RL, Beart J, Marger M, Wang M et al. (2000) Are nonspecific practice guidelines potentially harmful? A randomized comparison of the effect of nonspecific versus specific guidelines on physician decision making. Health Serv Res 34: 1429-1448. PubMed: 10737446. 10737446 PMC1975662

[5] Woolf SH, Grol R, Hutchinson A, Eccles M, Grimshaw J (1999) Clinical guidelines: potential benefits, limitations, and harms of clinical guidelines. BMJ 318: 527-530. doi:10.1136/bmj.318.7182.527. PubMed: 10024268. 10024268 PMC1114973

[6] Grimshaw J, Eccles M, Tetroe J (2004) Implementing clinical guidelines: current evidence and future implications. J Contin Educ Health Prof 24(Suppl 1): S31-S37. doi:10.1002/chp.1340240106. PubMed: 15712775. 15712775

[7] Grimshaw JM, Thomas RE, MacLennan G, Fraser C, Ramsay CR et al. (2004) Effectiveness and efficiency of guideline dissemination and implementation strategies. Health Technol Assess 8: iii-iiv, 1-72 PubMed: 14960256. 10.3310/hta8060 14960256

[8] Medves J, Godfrey C, Turner C, Paterson M, Harrison M et al. (2010) Systematic review of practice guideline dissemination and implementation strategies for healthcare teams and team-based practice. Int J Evid Based Healthc 8: 79-89. doi:10.1111/j.1479-6988.2010.00166.x. PubMed: 20923511. 20923511

[9] Hakkennes S, Dodd K (2008) Guideline implementation in allied health professions: a systematic review of the literature. Qual Saf Health Care 17: 296-300. doi:10.1136/qshc.2007.023804. PubMed: 18678729. 18678729

[10] AGREE Collaboration

[11] Burgers JS, Cluzeau FA, Hanna SE, Hunt C, Grol R (2003) Characteristics of high-quality guidelines: evaluation of 86 clinical guidelines developed in ten European countries and Canada. Int J Technol Assess Health Care 19: 148-157. PubMed: 12701947. 12701947 10.1017/s026646230300014x

[12] Grol R, Cluzeau FA, Burgers JS (2003) Clinical practice guidelines: towards better quality guidelines and increased international collaboration. Br J Cancer 89(Suppl 1): S4-S8. doi:10.1038/sj.bjc.6601077. PubMed: 12915896. 12915896 PMC2753001

[13] Shaneyfelt TM, Mayo-Smith MF, Rothwangl J (1999) Are guidelines following guidelines? The methodological quality of clinical practice guidelines in the peer-reviewed medical literature. JAMA 281: 1900-1905. doi:10.1001/jama.281.20.1900. PubMed: 10349893. 10349893

[14] Grilli R, Magrini N, Penna A, Mura G, Liberati A (2000) Practice guidelines developed by specialty societies: the need for a critical appraisal. Lancet 355: 103-106. doi:10.1016/S0140-6736(99)02171-6. PubMed: 10675167. 10675167

[15] Kryworuchko J, Stacey D, Bai N, Graham ID (2009) Twelve years of clinical practice guideline development, dissemination and evaluation in Canada (1994 to 2005). Implement Sci 4: 49. doi:10.1186/1748-5908-4-49. PubMed: 19656384. 19656384 PMC2731072

[16] Alonso-Coello P, Irfan A, Solà I, Gich I, Delgado-Noguera M et al. (2010) The quality of clinical practice guidelines over the last two decades: a systematic review of guideline appraisal studies. Qual Saf Health Care 19: e58. doi:10.1136/qshc.2010.042077. PubMed: 21127089. 21127089

[17] Kung J, Miller RR, Mackowiak PA (2012) Failure of clinical practice guidelines to meet institute of medicine standards: two more decades of little, if any. Progress - Arch Intern Med 172: 1628-1633. 23089902 10.1001/2013.jamainternmed.56

[18] Broedl UC, Geiss HC, Parhofer KG (2003) Comparison of current guidelines for primary prevention of coronary heart disease: risk assessment and lipid-lowering therapy. J Gen Intern Med 18: 190-195. doi:10.1046/j.1525-1497.2003.20207.x. PubMed: 12648250. 12648250 PMC1494828

[19] Friedman SE, Palac RT, Zlotnick DM, Chobanian MC, Costa SP (2011) A call to action: variability in guidelines for cardiac evaluation before renal transplantation. Clin J Am Soc Nephrol 6: 1185-1191. doi:10.2215/CJN.09391010. PubMed: 21511835. 21511835 PMC3087787

[20] Gillois P, Claudot F, Chatellier G, Kohler F, Jaulent MC (2006) Comparison of the impact of cardiovascular guidelines on a working population. Stud Health Technol Inform 124: 639-644. PubMed: 17108588. 17108588

[21] Kelly AM, Druda D (2008) Comparison of size classification of primary spontaneous pneumothorax by three international guidelines: a case for international consensus? Respir Med 102: 1830-1832. doi:10.1016/j.rmed.2008.07.026. PubMed: 18789858. 18789858

[22] Kyoong A, Mol S, Guy P, Finlay P, Strauss BJ et al. (2006) Comparison of Australian and international guidelines for grading severity of chronic obstructive pulmonary disease. Intern Med J 36: 506-512. doi:10.1111/j.1445-5994.2006.01142.x. PubMed: 16866655. 16866655

[23] Manuel DG, Kwong K, Tanuseputro P, Lim J, Mustard CA et al. (2006) Effectiveness and efficiency of different guidelines on statin treatment for preventing deaths from coronary heart disease: modelling study. BMJ 332: 1419 Available online at: doi:10.1136/bmj.38849.487546.DE. PubMed: 16737980. 16737980 10.1136/bmj.38849.487546.DE PMC1479685

[24] Sheehy AM, Flood GE, Tuan WJ, Liou JI, Coursin DB et al. (2010) Analysis of guidelines for screening diabetes mellitus in an ambulatory population. Mayo Clin Proc 85: 27-35. doi:10.4065/mcp.2010.0469. PubMed: 20042558. 20042558 PMC2800288

[25] Von Eckardstein A, Schulte H, Assmann G (2005) Comparison of international recommendations for the recognition of asymptomatic high risk patients for a heart attack in Germany [German]. Z Kardiol 94: 52-60. doi:10.1007/s00392-005-0150-4. PubMed: 15668832. 15668832

[26] Yu HR, Niu CK, Kuo HC, Tsui KY, Wu CC et al. (2010) Comparison of the Global Initiative for Asthma guideline-based asthma control measure and the Childhood Asthma Control Test in evaluating asthma control in children. Pediatr Neonatol 51: 273-278. doi:10.1016/S1875-9572(10)60053-8. PubMed: 20951357. 20951357

[27] Boyd CM, Darer J, Boult C, Fried LP, Boult L et al. (2005) Clinical practice guidelines and quality of care for older patients with multiple comorbid diseases: implications for pay for performance. JAMA 294: 716-724. doi:10.1001/jama.294.6.716. PubMed: 16091574. 16091574

[28] Mutasingwa DR, Ge H, Upshur RE (2011) How applicable are clinical practice guidelines to elderly patients with comorbidities? Can Fam Physician 57: e253-e262. PubMed: 21753084. 21753084 PMC3135464

[29] Fortin M, Contant E, Savard C, Hudon C, Poitras ME et al. (2011) Canadian guidelines for clinical practice: an analysis of their quality and relevance to the care of adults with comorbidity. BMC Fam Pract 12: 74. doi:10.1186/1471-2296-12-74. PubMed: 21752267. 21752267 PMC3146414

[30] Cox L, Kloseck M, Crilly R, McWilliam C, Diachun L (2011) Underrepresentation of individuals 80 years of age and older in chronic disease clinical practice guidelines. Can Fam Physician 57: e263-e269. PubMed: 21753085. 21753085 PMC3135465

[31] Chauhan SP, Berghella V, Sanderson M, Magann EF, Morrison JC (2006) American College of Obstetricians and Gynecologists practice bulletins: an overview. Am J Obstet Gynecol 194: 1564-1572. doi:10.1016/j.ajog.2006.03.001. PubMed: 16731072. 16731072

[32] Khan AR, Khan S, Zimmerman V, Baddour LM, Tleyjeh IM (2010) Quality and strength of evidence of the Infectious Diseases Society of America clinical practice guidelines. Clin Infect Dis 51: 1147-1156. doi:10.1086/656735. PubMed: 20946067. 20946067

[33] Lee DH, Vielemeyer O (2011) Analysis of overall level of evidence behind Infectious Diseases Society of America practice guidelines. Arch Intern Med 171: 18-22. PubMed: 21220656. 21220656 10.1001/archinternmed.2010.482

[34] McAlister FA, Van Diepen S, Padwal RS, Johnson JA, Majumdar SR (2007) How evidence-based are the recommendations in evidence-based guidelines? PLOS Med 4: 1325-1332. 10.1371/journal.pmed.0040250 PMC1939859 17683197

[35] Moyer VA, Butler M (2004) Gaps in the evidence for well-child care: a challenge to our profession. Pediatrics 114: 1511-1521. doi:10.1542/peds.2004-1076. PubMed: 15574609. 15574609

[36] Detsky AS (2006) Sources of bias for authors of clinical practice guidelines. CMAJ 175: 1033, 17060643. 17060643 10.1503/cmaj.061181 PMC1609150

[37] Shaneyfelt TM, Centor RM (2009) Reassessment of clinical practice guidelines: go gently into that good night. JAMA 301: 868-869. doi:10.1001/jama.2009.225. PubMed: 19244197. 19244197

[38] Sniderman AD, Furberg CD (2009) Why guideline-making requires reform. JAMA 301: 429-431. doi:10.1001/jama.2009.15. PubMed: 19176446. 19176446

[39] Williams MJ, Kevat DA, Loff B (2011) Conflict of interest guidelines for clinical guidelines. Med J Aust 195: 442-445. doi:10.5694/mja10.11130. PubMed: 22004385. 22004385

[40] Rosumeck S, Sporbeck B, Rzany B, Nast A (2011) Disclosure of potential conflicts of interest in dermatological guidelines in Germany: an analysis; status quo and quo vadis [German]. J Dtsch Dermatol Ges 9: 297-304. doi:10.1111/j.1610-0387.2011.07615_suppl.x. PubMed: 21439013. 21439013

[41] Coyne DW (2007) Influence of industry on renal guideline development. Clin J Am Soc Nephrol 2: 3-7. PubMed: 17699377. 17699377 10.2215/CJN.02170606

[42] Qaseem A, Forland F, Macbeth F, Ollenschläger G, Phillips S et al. (2012) Guidelines International Network: toward international standards for clinical practice guidelines. Ann Intern Med 156: 525-531. doi:10.7326/0003-4819-156-7-201204030-00009. PubMed: 22473437. 22473437

[43] World Health Organization (2003) Guidelines for WHO guidelines. Available: http://whqlibdoc.who.int/hq/2003/EIP_GPE_EQC_2003_1.pdf. Accessed 18 November 2011

[44] National Institute for Health and Clinical Excellence (2009) The guidelines manual. Available: http://www.nice.org.uk/guidelinesmanual. Accessed 19 October 2012

[45] Scottish Intercollegiate Guidelines Network (2011) SIGN 50: a guideline developer’s handbook. Edinburgh: SIGN Available: http://www.sign.ac.uk/pdf/sign50.pdf.

[46] American College of Cardiology Foundation and American Heart Association (2010) Methodology manual and policies from the ACCF/AHA Task Force on Practice Guidelines. Available: http://my.americanheart.org/idc/groups/ahamah-public/@wcm/@sop/documents/downloadable/ucm_319826.pdf. Accessed 19 October 2012

[47] Gronseth G, Moses Woodroffe L, Getchius TSD (2011) Clinical practice guideline process manual: 2011 edition. Available: http://tools.aan.com/globals/axon/assets/9023.pdf. Accessed 04 July 2013

[48] Baumann MH, Lewis SZ, Gutterman D (2007) ACCP evidence-based guideline development: a successful and transparent approach addressing conflict of interest, funding, and patient-centered recommendations. Chest 132: 1015-1024. doi:10.1378/chest.07-1271. PubMed: 17540835. 17540835

[49] Qaseem A, Snow V, Owens DK, Shekelle P (2010) The development of clinical practice guidelines and guidance statements of the American College of Physicians: summary of methods. Ann Intern Med 153: 194-199. doi:10.7326/0003-4819-153-3-201008030-00010. PubMed: 20679562. 20679562

[50] Deutsche Gesellschaft für Unfallchirurgie (2008); der Leitlinienentwicklung Methodik, der DGU. Available: http://www.dgu-online.de/qualitaet-sicherheit/leitlinien/methodik-der-leitlinienentwicklung-der-dgu.html. Accessed 02 July 2013

[51] Platz T, Quintern J (2009) Methodik der Leitlinien-Entwicklung der Leitlinien-Kommission der Deutschen Gesellschaft für Neurorehabilitation. Neurologie und Rehabilitation 15: 75-80.

[52] Rosenfeld RM, Shiffman RN (2006) Clinical practice guidelines: a manual for developing evidence-based guidelines to facilitate performance measurement and quality improvement. Otolaryngol Head Neck Surg 135(4 Suppl): S1-S28. doi:10.1016/j.otohns.2006.05.733. PubMed: 17023260. 17023260

[53] Woolf S, Schünemann HJ, Eccles MP, Grimshaw JM, Shekelle P (2012) Developing clinical practice guidelines: types of evidence and outcomes; values and economics, synthesis, grading, and presentation and deriving recommendations. Implement Sci 7: 61. doi:10.1186/1748-5908-7-61. PubMed: 22762158. 22762158 PMC3436711

[54] Shekelle P, Woolf S, Grimshaw JM, Schünemann HJ, Eccles MP (2012) Developing clinical practice guidelines: reviewing, reporting, and publishing guidelines; updating guidelines; and the emerging issues of enhancing guideline implementability and accounting for comorbid conditions in guideline development. Implement Sci 7: 62. doi:10.1186/1748-5908-7-62. PubMed: 22762242. 22762242 PMC3503794

[55] Eccles MP, Grimshaw JM, Shekelle P, Schünemann HJ, Woolf S (2012) Developing clinical practice guidelines: target audiences, identifying topics for guidelines, guideline group composition and functioning and conflicts of interest. Implement Sci 7: 60. doi:10.1186/1748-5908-7-60. PubMed: 22762776. 22762776 PMC3523009

[56] Turner T, Misso M, Harris C, Green S (2008) Development of evidence-based clinical practice guidelines (CPGs): comparing approaches. Implement Sci 3: 45. doi:10.1186/1748-5908-3-45. PubMed: 18954465. 18954465 PMC2584093

[57] AGREE Next Steps Consortium (2009) Appraisal of guidelines for research and evaluation II: AGREE II instrument. Available: http://www.agreetrust.org/index.aspx?o=1397. Accessed 02 December 2011

[58] Graham ID, Calder LA, Hébert PC, Carter AO, Tetroe JM (2000) A comparison of clinical practice guideline appraisal instruments. Int J Technol Assess Health Care 16: 1024-1038. doi:10.1017/S0266462300103095. PubMed: 11155826. 11155826

[59] Vlayen J, Aertgeerts B, Hannes K, Sermeus W, Ramaekers D (2005) A systematic review of appraisal tools for clinical practice guidelines: multiple similarities and one common deficit. Int J Qual Health Care 17: 235-242. doi:10.1093/intqhc/mzi027. PubMed: 15743883. 15743883

[60] Cluzeau FA, Littlejohns P, Grimshaw JM, Feder G, Moran SE (1999) Development and application of a generic methodology to assess the quality of clinical guidelines. Int J Qual Health Care 11: 21-28. doi:10.1093/intqhc/11.1.21. PubMed: 10411286. 10411286

[61] Hayward RSA, Wilson MC, Tunis SR, Bass EB, Guyatt G (1995) Users' guides to the medical literature; VIII; how to use clinical practice Guidelines; A; are the recommendations valid? JAMA 274: 570-574 10.1001/jama.274.7.570 7629987

[62] Wilson MC, Hayward RS, Tunis SR, Bass EB, Guyatt G (1995) Users' guides to the medical literature: VIII; how to use clinical practice Guidelines; B; what are the recommendations and will they help you in caring for your patients? JAMA 274: 1630-1632 10.1001/jama.274.7.570 7629987

[63] Field MJ, Lohr KN (1992) Guidelines for clinical practice: from development to use. Washington: National Academy Press. 25121254

[64] ADAPTE Collaboration (2010) Guideline adaption: a resource toolkit; version 2.0. Available: http://www.g-i-n.net/document-store/adapte-resource-toolkit-guideline-adaptation-version-2. Accessed 30 April 2012

[65] Arbeitsgemeinschaft der Wissenschaftlichen Medizinischen Fachgesellschaften, Ärztliches Zentrum für Qualität in der Medizin (2008) Deutsches Instrument zur methodischen Leitlinien-Bewertung (DELBI): Fassung 2005/2006 + Domäne 8 (2008). Available: http://www.aezq.de/mdb/edocs/pdf/literatur/delbi-fassung-2005-2006-domaene-8-2008.pdf. Accessed 25 November 2011

[66] Chong CA, Chen IJ, Naglie G, Krahn MD (2009) How well do guidelines incorporate evidence on patient preferences? J Gen Intern Med 24: 977-982. doi:10.1007/s11606-009-0987-8. PubMed: 19387746. 19387746 PMC2710487

[67] Kashyap N, Dixon J, Michel G, Brandt C, Shiffman RN (2011). Glia: Guideline Implementability Appraisal V. 2: 0 Available: http://gem.med.yale.edu/glia/doc/GLIA_v2.pdf. Accessed 28 December 2011 10.1186/1472-6947-5-23 PMC1190181 16048653

[68] Burgers JS, Bailey JV, Klazinga NS, Van Der Bij AK, Grol R et al. (2002) Inside guidelines: comparative analysis of recommendations and evidence in diabetes guidelines from 13 countries. Diabetes Care 25: 1933-1939. doi:10.2337/diacare.25.11.1933. PubMed: 12401735. 12401735

[69] Raine R, Sanderson C, Hutchings A, Carter S, Larkin K et al. (2004) An experimental study of determinants of group judgments in clinical guideline development. Lancet 364: 429-437. doi:10.1016/S0140-6736(04)16766-4. PubMed: 15288741. 15288741

[70] Pagliari C, Grimshaw J, Eccles M (2001) The potential influence of small group processes on guideline development. J Eval Clin Pract 7: 165-173. doi:10.1046/j.1365-2753.2001.00272.x. PubMed: 11489041. 11489041

[71] Pagliari C, Grimshaw J (2002) Impact of group structure and process on multidisciplinary evidence-based guideline development: an observational study. J Eval Clin Pract 8: 145-153. doi:10.1046/j.1365-2753.2002.00333.x. PubMed: 12180363. 12180363

[72] Fretheim A, Schünemann HJ, Oxman AD (2006) Improving the use of research evidence in guideline development: 3; group composition and consultation process. Health Res Policy Syst 4: 15. 10.1186/1478-4505-4-15 PMC1702349 17134482

[73] Moreira T, May C, Mason J, Eccles M (2006) A new method of analysis enabled a better understanding of clinical practice guideline development processes. J Clin Epidemiol 59: 1199-1206. doi:10.1016/j.jclinepi.2005.08.021. PubMed: 17027431. 17027431

[74] Gardner B, Davidson R, McAteer J, Michie S (2009) A method for studying decision-making by guideline development groups. Implement Sci 4: 48. doi:10.1186/1748-5908-4-48. PubMed: 19656366. 19656366 PMC2731071

[75] Zuiderent-Jerak T, Forland F, Macbeth F (2012) Guidelines should reflect all knowledge, not just clinical trials. BMJ 345: e6702. doi:10.1136/bmj.e6702. PubMed: 23043093. 23043093

[76] Als-Nielsen B, Chen W, Gluud C, Kjaergard LL (2003) Association of funding and conclusions in randomized drug trials: a reflection of treatment effect or adverse events? JAMA 290: 921-928. doi:10.1001/jama.290.7.921. PubMed: 12928469. 12928469

[77] Lexchin J, Bero LA, Djulbegovic B, Clark O (2003) Pharmaceutical industry sponsorship and research outcome and quality: systematic review. BMJ 326: 1167-1170. doi:10.1136/bmj.326.7400.1167. PubMed: 12775614. 12775614 PMC156458

[78] Boyd EA, Bero LA (2000) Assessing faculty financial relationships with industry: a case study. JAMA 284: 2209-2214. doi:10.1001/jama.284.17.2209. PubMed: 11056592. 11056592

[79] Campbell EG (2007) Doctors and drug companies: scrutinizing influential relationships. N Engl J Med 357: 1796-1797. doi:10.1056/NEJMp078141. PubMed: 17978288. 17978288

[80] Jacobs AK, Lindsay BD, Bellande BJ, Fonarow GC, Nishimura RA et al. (2004) Task force 3: Disclosure of relationships with commercial interests; policy for educational activities and publications. J Am Coll Cardiol 44: 1736-1740. doi:10.1016/j.jacc.2004.08.040. PubMed: 15489117. 15489117

[81] American Professional Wound Care Association (2010) SELECT: evaluation and implementation of clinical practice guidelines; a guidance document from the American Professional Wound Care Association. Adv Skin Wound Care 23: 161-168. PubMed: 20299842. 20299842 10.1097/01.ASW.0000363529.93253.dd

[82] Baxter NN, Palda VA (2003) Guidelines for colorectal surgery. Semin Colon Rectal Surg 14: 19-25. doi:10.1053/scrs.2003.127416.

[83] Bundesärztekammer, Kassenärztliche Bundesvereinigung (1997) Beurteilungskriterien für Leitlinien in der medizinischen Versorgung: Beschlüsse der Vorstände von Bundesärztekammer und Kassenärztlicher Bundesvereinigung, Juni 1997. Dtsch Arztebl 94: A2154-A2155.

[84] Chou R (2008) Using evidence in pain practice: part II; interpreting and applying systematic reviews and clinical practice Guidelines Pain Med 9: 531-541

[85] Chou R (2008) Using evidence in pain practice: part I; assessing quality of systematic reviews and clinical practice Guidelines Pain Med 9: 518-530 10.1111/j.1526-4637.2008.00422_2.x 18346061

[86] Fields SD (2000) Clinical practice guidelines: finding and appraising useful, relevant recommendations for geriatric care. Geriatrics 55: 59-63. PubMed: 10659074. 10659074

[87] Foy R, MacLennan G, Grimshaw J, Penney G, Campbell M et al. (2002) Attributes of clinical recommendations that influence change in practice following audit and feedback. J Clin Epidemiol 55: 717-722. doi:10.1016/S0895-4356(02)00403-1. PubMed: 12160920. 12160920

[88] Guyatt G, Hayward RS, Richardson WS, Green L, Wilson MC et al. (2002) Moving from evidence to action. In: Guyatt G Drummond R Users’ guides to the medical literature. Chicago: AMA Press pp. 175-199.

[89] Liddle J, Williamson M, Irwig L (1996) Method for Evaluation Research Guidelines Evidence (MERGE). Sydney: New South Wales Department of Health Available: http://www0.health.nsw.gov.au/pubs/1996/pdf/mergetot.pdf.

[90] Linskey ME (2010) Defining excellence in evidence-based medicine clinical practice guidelines. Clin Neurosurg 57: 28-37. PubMed: 21280492. 21280492

[91] Marshall JK (2000) A critical approach to clinical practice guidelines. Can J Gastroenterol 14: 505-509. PubMed: 10888731. 10888731 10.1155/2000/302785

[92] Nonino F, Liberati A (2004) Essential requirements for practice guidelines at national and local levels. Neurol Sci 25: 2-7. doi:10.1007/s10072-004-0217-7. PubMed: 15060808. 15060808

[93] Sanderlin BW, AbdulRahim N (2007) Evidence-based medicine, part 6: an introduction to critical appraisal of clinical practice guidelines. J Am Osteopath Assoc 107: 321-324. PubMed: 17785690. 17785690

[94] Veale B, Weller D, Silagy C (1999) Clinical practice guidelines and Australian general practice: contemporary issues. Aust Fam Physician 28: 744-749. PubMed: 10431441. 10431441

[95] Warriner RA, Carter MJ (2011) The current state of evidence-based protocols in wound care. Plast Reconstr Surg 127(Suppl 1): 144S-153S. doi:10.1097/PRS.0b013e31820023dc. PubMed: 21200285. 21200285

[96] Woolf SH (1995) Practice guidelines: what the family physician should know. Am Fam Physician 51: 1455-1463. PubMed: 7732947. 7732947

[97] American Psychological Association (2002) Criteria for evaluating treatment guidelines. Am Psychol 57: 1052-1059. doi:10.1037/0003-066X.57.12.1052. PubMed: 12617064. 12617064

[98] Fretheim A, Williams JW Jr, Oxman AD, Herrin J (2002) The relation between methods and recommendations in clinical practice guidelines for hypertension and hyperlipidemia. J Fam Pract 51: 963-968. PubMed: 12485552. 12485552

[99] Hart RG, Bailey RD (2002) An assessment of guidelines for prevention of ischemic stroke. Neurology 59: 977-982. doi:10.1212/WNL.59.7.977. PubMed: 12374137. 12374137

[100] Hasenfeld R, Shekelle PG (2003) Is the methodological quality of guidelines declining in the US? Comparison of the quality of US Agency for Health Care Policy and Research (AHCPR) guidelines with those published subsequently. Qual Saf Health Care 12: 428-434. doi:10.1136/qhc.12.6.428. PubMed: 14645758. 14645758 PMC1758044

[101] Hindley C, Hinsliff SW, Thomson AM (2005) Developing a tool to appraise fetal monitoring guidelines for women at low obstetric risk. J Adv Nurs 52: 307-314. doi:10.1111/j.1365-2648.2005.03593.x. PubMed: 16194184. 16194184

[102] Kulig M, Schulte E, Willich S (2003) Comparing methodological quality and consistency of international guidelines for the management of patients with chronic heart failure. Eur J Heart Fail 5: 327-335. doi:10.1016/S1388-9842(03)00040-0. PubMed: 12798831. 12798831

[103] Pentheroudakis G, Stahel R, Hansen H, Pavlidis N (2008) Heterogeneity in cancer guidelines: should we eradicate or tolerate? Ann Oncol 19: 2067-2078. doi:10.1093/annonc/mdn418. PubMed: 18662954. 18662954 PMC2733109

[104] Sanders GD, Nease RF Jr, Owens DK (2000) Design and pilot evaluation of a system to develop computer-based site-specific practice guidelines from decision models. Med Decis Making 20: 145-159. doi:10.1177/0272989X0002000201. PubMed: 10772353. 10772353

[105] Savoie I, Kazanjian A, Bassett K (2000) Do clinical practice guidelines reflect research evidence? J Health Serv Res Policy 5: 76-82. PubMed: 10947551. 10947551 10.1177/135581960000500204

[106] Ward JE, Grieco V (1996) Why we need guidelines for guidelines: a study of the quality of clinical practice guidelines in Australia. Med J Aust 165: 574-576. PubMed: 8941245. 8941245 10.5694/j.1326-5377.1996.tb138645.x

[107] Calder L, Hébert P, Carter A, Gaham I (1997) Review of published recommendations and guidelines for the transfusion of allogenieic red blood cell and plasma. Can Med Assoc J 156(11 Suppl): S1-S8.9347786

[108] Hargrove P, Griffer M, Lund B (2008) Procedures for using clinical practice guidelines. Lang Speech Hear Serv Sch 39: 289-302. doi:10.1044/0161-1461(2008/028). PubMed: 18596287. 18596287

[109] Burls A (2010) AGREE II: improving the quality of clinical care. Lancet 376: 1128-1129. doi:10.1016/S0140-6736(10)61034-3. PubMed: 20599263. 20599263

[110] Watine J, Friedberg B, Nagy E, Onody R, Oosterhuis W et al. (2006) Conflict between guideline methodologic quality and recommendation validity: a potential problem for practitioners. Clin Chem 52: 65-72. doi:10.1373/clinchem.2005.056952. PubMed: 16391328. 16391328

[111] Nuckols TK, Lim YW, Wynn BO, Mattke S, MacLean CH et al. (2008) Rigorous development does not ensure that guidelines are acceptable to a panel of knowledgeable providers. J Gen Intern Med 23: 37-44. doi:10.1007/s11606-007-0440-9. PubMed: 18030541. PMC2173921 18030541

[112] Brouwers MC, Kho ME, Browman GP, Burgers JS, Cluzeau F et al. (2010) AGREE II: advancing guideline development, reporting and evaluation in health care. CMAJ 182: E839-E842. doi:10.1503/cmaj.090449. PubMed: 20603348. 20603348 PMC3001530

[113] Field MJ, Lohr KN (1990) Clinical practice guidelines: directions for a new program. Washington: National Academy Press. 25144032

[114] AGREE Research Trust the AGREE A3 Research Team. Available: http://www.agreetrust.org/about-agree/agree-research-teams/agree-a3-research-team. Accessed 17 September 2012

[115] Institut für Qualität und Wirtschaftlichkeit im Gesundheitswesen [V06-07]: Erprobung einer Methode zur Bewertung der internen Validität von Leitlinienempfehlungen am Beispiel evidenzbasierter Leitlinien zur präoperativen Diagnostik; Rapid Report. Available: https://www.iqwig.de/v06-07-erprobung-einer-methode-zur-bewertung-der.986.html?tid=1347. Accessed 17 September 2012

[116] Hayward RSA, Wilson MC, Tunis SR, Bass EB, Rubin HR et al. (1993) More informative abstracts of articles describing clinical practice guidelines. Ann Intern Med 118: 731-737. doi:10.7326/0003-4819-118-9-199305010-00012. PubMed: 8460861. 8460861

[117] Guyatt GH, Sackett DL, Sinclair JC, Hayward R, Cook DJ, et al. (1995) Users' guides to the medical literature: IX; a method for grading health care recommendations. JAMA 274: 1800-1804. 10.1001/jama.274.22.1800 7500513

[118] Atkins D, Best D, Briss PA, Eccles M, Falck-Ytter Y et al. (2004) Grading quality of evidence and strength of recommendations. BMJ 328: 1490. doi:10.1136/bmj.328.7454.1490. PubMed: 15205295. 15205295 PMC428525

[119] Cook DJ, Ellrodt AG, Calvin J, Levy MM (1998) How to use practice guidelines in the intensive care unit: diagnosis and management of unstable angina. Crit Care Med 26: 599-606. doi:10.1097/00003246-199803000-00038. PubMed: 9504592. 9504592

[120] Grilli R, Lomas J (1994) Evaluating the message: the relationship between compliance rate and the subject of a practice guideline. Med Care 32: 202-213. doi:10.1097/00005650-199403000-00002. PubMed: 8145598. 8145598

[121] Grol R, Dalhuijsen J, Thomas S, Veld C, Rutten G et al. (1998) Attributes of clinical guidelines that influence use of guidelines in general practice: observational study. BMJ 317: 858-861. doi:10.1136/bmj.317.7162.858. PubMed: 9748183. 9748183 PMC31096

[122] Cluzeau F, Littlejohns P, Grimshaw J, Feder G (1997) Appraisal instrument for clinical Guidelines. London: St George’s Hospital Medical School

[123] Solberg LI, Brekke ML, Fazio CJ, Fowles J, Jacobsen DN et al. (2000) Lessons from experienced guideline implementers: attend to many factors and use multiple strategies. Jt Comm J Qual Improv 26: 171-188. PubMed: 10749003. 10749003 10.1016/s1070-3241(00)26013-6

[124] Thorson T, Mäkelä M (1999) Changing professional practice: theory and practice of clinical guidelines implementation. Copenhagen: Danish Institute for Health Services Research and Development.

[125] Pinsky LE, Deyo RA (2000) Clinical guidelines: a strategy for translating evidence into practice. In: Geyman JP Deyo RA Ramsey SD Evidence-based clinical practice: concepts and approaches. Boston: Butterworth Heinemann pp. 119-123.

[126] Sackett DL, Straus SE, Richardson WS, Rosenberr W, Haynes RB (2000) Evidence-based medicine: how to practice and teach EbM. Edinburgh: Churchill Livingston.

[127] Snowball R (2005) Critical appraisal of clinical guidelines. In: Dawes M Davies P Gray A Evidence based practice: a primer for health professionals. Edinburgh: Elsevier Churchill Livingstone pp. 127-131.

[128] Nicholson D (2002) Practice guidelines: a strategy for translating evidence into practice. In: Law M Evidence based rehabilitation: a guide to practice. Thorofare: Slack pp. 193-219.

[129] American Medical Association (1990) Attributes to guide the development of practice parameters. Chicago: AMA.

[130] Hayward RSA (1993) Initiating, conducting and maintaining guideline development programs. Can Med Assoc J 148: 507-512. 8431814 PMC1490514

[131] Agency for Healthcare Research and Quality (2011) National Guidelines Clearinghouse. Available: http://www.guideline.gov. Accessed 16 December 2011 10.1080/15360280802537332 21923316

[132] American Gastroenterological Association (1995) American Gastroenterological Association policy statement on the use of medical practice guidelines by managed care organizations and insurance carriers. Gastroenterology 108: 925-926. doi:10.1016/0016-5085(95)28006-5. PubMed: 7875497. 7875497

[133] Canadian Medical Association (1994) Guidelines for Canadian clinical practice guidelines. Ottawa: CMA.

[134] Mottur-Pilson C (1995) Internists' evaluation of guidelines: the IMCARE Practice Guidelines. Netw - Int J Qual Health Care 7: 31-37. doi:10.1016/1353-4505(94)00049-N. 7640916

[135] Sonnad S, McDonald TW, Nease RF, Oleske J, Owens DK (1993) An evaluation of the methodology of guidelines for zidovudine therapy in HIV disease. Med Decis Making 13: 398.

[136] Hadorn DC, Baker D (1994) Development of the AHCPR-sponsored heart failure guideline: methodologic and procedural issues. Jt Comm J Qual Improv 20: 539-547. PubMed: 7842059. 7842059 10.1016/s1070-3241(16)30099-2

[137] Shiffman RN, Karras BT, Agrawal A, Chen R, Marenco L et al. (2000) GEM: a proposal for a more comprehensive guideline document model using XML. J Am Med Inform Assoc 7: 488-498. doi:10.1136/jamia.2000.0070488. PubMed: 10984468. 10984468 PMC79044

