# Supplementary material for: Appraisal Tools for Clinical Practice Guidelines: A Systematic Review
Source: PLoS One. 2013 Dec 9;8(12):e82915. doi: 10.1371/journal.pone.0082915 (PMC3857289; doi:10.1371/journal.pone.0082915)
Supplement: File S1 — Search strategy. (PDF) [file pone.0082915.s002.pdf]

Supporting information 1 – Search strategy

1. EMBASE

Search interface: Ovid

- Embase 1980 to 2011 May 09

#	Searches
1	*Practice guideline/
2	(guideline or guidelines).ti.
3	1 or 2
4	(appraisal or appraised).ab.
5	(apprais* or quality or evaluation or evaluated or analysis or analysing).ti.
6	or/4-5
7	(review or study).ab,ti.
8	evidence-based.ti.
9	(grade or grading or agree).ti,ab.
10	Evidence based medicine/
11	Health care quality/
12	Health care policy/
13	Systematic review/
14	Review/
15	Comparative study/
16	Controlled study/
17	or/7-16
18	and/3,6,17
19	18 not medline*.cr.
20	limit 19 to yr="1995 -Current"
21	limit 20 to (english or german)

2. MEDLINE

Search interface: Ovid

- Ovid MEDLINE(R) 1948 to April Week 4 2011
- Ovid MEDLINE(R) Daily Update May 09, 2011
- Ovid MEDLINE(R) In-Process & Other Non-Indexed Citations May 09, 2011

#	Searches
1	*Practice Guidelines as Topic/
2	(guideline or guidelines).ti.
3	1 or 2
4	(appraisal or appraised).ab.
5	(apprais* or quality or evaluation or evaluated or analysis or analysing or comparison or valid or attributes).ti.
6	Practice Guidelines as Topic/st
7	Physician's Practice Patterns/st
8	or/4-7
9	(review or study).ab,ti.
10	evidence-based.ti.
11	(grade or grading or agree).ti,ab.
12	Evidence-Based Medicine/
13	Reproducibility of Results/
14	Quality Assurance, Health Care/
15	Evaluation Studies as Topic/
16	Quality of Health Care/
17	Guideline Adherence/
18	or/9-17
19	and/3,8,18
20	limit 19 to yr="1995 -Current"
21	limit 20 to (english or german)

3. PubMed

Search interface: NLM

- PubMed - as supplied by publisher
- PubMed - in process
- PubMed – OLDMEDLINE
- PubMed - pubmednotmedline

Search	Most Recent Queries
#1	Search guideline[TI] OR guidelines[TI]
#2	Search apprais*[TIAB]
#3	Search quality[TI] OR evaluation[TI] OR evaluated[TI] OR analysis[TI] OR analysing[TI] OR comparison[TI] OR valid[TI] OR attributes[TI]
#4	Search review[TIAB] or study[TIAB]
#5	Search evidence-based[TI]
#6	Search grade[TIAB] or grading[TIAB] or agree[TIAB]
#7	Search #1 AND (#2 OR #3) AND (#4 OR #5 OR #6)
#8	Search #7 NOT medline[sb]
#9	Search Limits: Publication Date from 1995 to 2011
#10	Search #8 AND #9 Limits: Publication Date from 1995 to 2011

4. The Cochrane Library

Search interface: Wiley

- Cochrane Database of Systematic Reviews (Cochrane Reviews), Issue 4 2011
- Database of Abstracts of Reviews of Effects (Other Reviews), Issue 2, 2011
- Health Technology Assessment Database (Technology Assessments), Issue 2, 2011
- NHS Economic Evaluation Database, Issue 2, 2011
- Cochrane Methodology Register, Issue 2, 2011

ID	Search
#1	MeSH descriptor Practice Guidelines as Topic, this term only
#2	(guideline or guidelines):ti
#3	(#1 OR #2)
#4	apprais*:ab,ti
#5	(quality or evaluation or evaluated or analysis or analysing or comparison or valid or attributes):ti
#6	MeSH descriptor Practice Guidelines as Topic explode all trees with qualifier: ST
#7	MeSH descriptor Physician's Practice Patterns explode all trees with qualifier: ST
#8	(#4 OR #5 OR #6 OR #7)
#9	(review or study):ab,ti
#10	evidence-based:ti
#11	(grade or grading or agree):ti,ab
#12	MeSH descriptor Evidence-Based Medicine, this term only
#13	MeSH descriptor Reproducibility of Results, this term only
#14	MeSH descriptor Quality Assurance, Health Care, this term only
#15	MeSH descriptor Evaluation Studies as Topic, this term only
#16	MeSH descriptor Quality of Health Care, this term only
#17	MeSH descriptor Guideline Adherence explode all trees
#18	(#9 OR #10 OR #11 OR #12 OR #13 OR #14 OR #15 OR #16 OR #17)
#19	(#3 AND #8 AND #18)
#20	(#19), from 1995 to 2011
