# Supplementary material for: Appraisal Tools for Clinical Practice Guidelines: A Systematic Review
Source: PLoS One. 2013 Dec 9;8(12):e82915. doi: 10.1371/journal.pone.0082915 (PMC3857289; doi:10.1371/journal.pone.0082915)
Supplement: File S2 — Excluded studies (ordered by reasons for exclusion). (PDF) [file pone.0082915.s003.pdf]

Supporting information 2 – Excluded studies (ordered by reasons for exclusion)

Not an appraisal tool

1. Advani A, Shahar Y, Musen MA (2001) Medical quality assessment by scoring adherence to guideline intentions. Proc AMIA Symp: 2-6.

2. Al-Ansary L, Alkhenizan A (2004) Towards evidence-based clinical practice guidelines in Saudi Arabia. Saudi Med J 25: 1555-1558.

3. Ärztliche Zentralstelle Qualitätssicherung (2000) Leitlinien-Clearing-Bericht "Hypertonie". Available: http://www.leitlinien.de/mdb/edocs/pdf/schriftenreihe/schriftenreihe5.pdf. Accessed 01 July 2013.

4. Blasi F (2006) History and critical analysis of the guidelines for the treatment of lower respiratory tract infections. J Chemother 18: 32-34.

5. Bouaud J, Seroussi B, Falcoff H, Julien J, Simon C, et al. (2009) Consequences of the verification of completeness in clinical practice guideline modeling: a theoretical and empirical study with hypertension. AMIA Annu Symp Proc 2009: 60-64.

6. Brok J, Greisen G, Jacobsen T, Gluud L, Gluud C (2007) Agreement between Cochrane Neonatal Group reviews and clinical guidelines for newborns at a Copenhagen University Hospital: a cross-sectional study. Acta Paediatr 96: 39-43.

7. Burgers JS (2006) Guideline quality and guideline content: are they related? Clin Chem 52: 3-4.

8. Burgers JS, Van Everdingen JJ (2004) Beyond the evidence in clinical guidelines. Lancet 364: 392-393.

9. Burnand B, Vader JP, Froehlich F, Dupriez K, Larequi-Lauber T, et al. (1998) Reliability of panel-based guidelines for colonoscopy: an international comparison. Gastrointest Endosc 47: 162-166.

10. Chauhan SP, Berghella V, Sanderson M, Siddiqui D, Hendrix NW, et al. (2009) Randomized clinical trials behind level A recommendations in obstetric practice bulletins: compliance with CONSORT statement. Am J Perinatol 26: 69-80.

11. Chestnut JL (2003) Evaluating the quality of clinical practice guidelines. J Manipulative Physiol Ther 26: 208-209.

12. Choi J, Currie L, Wang D, Bakken S (2007) Encoding a clinical practice guideline using guideline interchange format: a case study of a depression screening and management guideline. Int J Med Inf 76(Suppl 2): S302-S307.

13. Cluzeau F (2009) Conflicting recommendations: let's not forget AGREE. BMJ 338: b407.

14. Cluzeau F, Littlejohns P, Grimshaw J, Hopkins A (1995) Appraising clinical guidelines and the development of criteria: a pilot study. J Interprof Care 9: 227-235.

15. Cluzeau FA, Littlejohns P (1999) Appraising clinical practice guidelines in England and Wales: the development of a methodologic framework and its application to policy. Jt Comm J Qual Improv 25: 514-521.

16. Cook DJ, Greengold NL, Ellrodt AG, Weingarten SR (1997) The relation between systematic reviews and practice guidelines. Ann Intern Med 127: 210-216.

17. Cutting KF, White RJ (2008) Quality assurance that will ensure robust and transparent guidelines. J Wound Care 17: 451.

18. Dans A, Dans L (2010) Appraising a tool for guideline appraisal (the AGREE II instrument). J Clin Epidemiol 63: 1281-1282.

19. Djulbegovic B, Hadley T (1998) Evaluating the quality of clinical guidelines: linking decisions to medical evidence. Oncology (Williston Park) 12: 310-314.

20. Egidi G (2008) Analysis of a planned guideline on diabetes treatment written by the German Diabetes Association (DDG): instructive arguments against often heard but wrong arguments in diabetes care [German]. Z Allgemeinmed 84: 451-456.

21. Entwistle M, Shiffman RN (2005) Turning guidelines into practice: making it happen with standards; part 1. Health Care and Informatics Review Online 9.

22. Fervers B, Burgers JS, Haugh M, Latreille J, Mlika-Cabanne N, et al. (2006) Adaptation of clinical guidelines: literature review and proposition for a framework and procedure. Int J Qual Health Care 18: 167-176.

23. Fervers B, Philip T, Browman GP (2002) Critical appraisal of the minimal clinical recommendations (MCR) of the European Society for Medical Oncology (ESMO): challenges for a European framework for the development of clinical practice guideline. Ann Oncol 13: 1507-1509.

24. Freeman M (2010) Clinical practice guidelines versus systematic reviews; which serve as the best basis for evidence-based spine medicine? Spine J 10: 512-513.

25. Gartlehner G, West SL, Lohr KN, Kahwati L, Johnson JG, et al. (2004) Assessing the need to update prevention guidelines: a comparison of two methods. Int J Qual Health Care 16: 399-406.

26. Gethin G (2009) Use of the AGREE tool will improve guideline implementation. J Wound Care 18: 40.

27. Graham ID, Calder LA, Hebert PC, Carter AO, Tetroe JM (2000) A comparison of clinical practice guideline appraisal instruments. Int J Technol Assess Health Care 16: 1024-1038.

28. Graham RP, James PA, Cowan TM (2000) Are clinical practice guidelines valid for primary care? J Clin Epidemiol 53: 949-954.

29. Harris JS, Mueller K, Low P, Peplowski B, Koziol-McLain J (2000) Suggested improvements in practice guidelines: market research to support clinical quality improvement. J Occup Environ Med 42: 377-384.

30. Hauser W, Ziehl S, Poltorak P, Grandt D (2005) Is an evidence-based therapy of inpatients with type 2 diabetes possible from studies used for formulating therapy guidelines? [German]. Dtsch Med Wochenschr 130: 1069-1073.

31. Hegarty K, Gunn J, Blashki G, Griffiths F, Dowell T, et al. (2009) How could depression guidelines be made more relevant and applicable to primary care? A quantitative and qualitative review of national guidelines. Br J Gen Pract 59: e149-e156.

32. Heiat A, Vaccarino V, Krumholz HM (2001) An evidence-based assessment of federal guidelines for overweight and obesity as they apply to elderly persons. Arch Intern Med 161: 1194-1203.

33. Hinsliff S, Hindley C, Thomson A (2004) A survey of regional guidelines for intrapartum electronic fetal monitoring in women at low obstetric risk. Midwifery 20: 345-357.

34. Hommersom A, Groot P, Balser M, Lucas P (2008) Formal methods for verification of clinical practice guidelines. Stud Health Technol Inform 139: 63-80.

35. Humpel N, Iverson D (2005) Review and critique of the quality of exercise recommendations for cancer patients and survivors. Support Care Cancer 13: 493-502.

36. James PA, Cowan TM, Graham RP, Majeroni BA, Fox CH, et al. (1997) Using a clinical practice guidelines to measure physician practice: translating a guideline for the management of heart failure. J Am Board Fam Pract 10: 206-212.

37. Keating J (2002) Evaluating the quality of clinical practice guidelines. J Manipulative Physiol Ther 25: 423.

38. Kent C (2001) Evaluating the quality of clinical practice guidelines. J Manipulative Physiol Ther 24: 612-618.

39. Keuken DG, Haafkens JA, Moerman CJ, Klazinga NS, Ter Riet G (2007) Attention to sex-related factors in the development of clinical practice guidelines. J Womens Health 16: 82-92.

40. Knottnerus JA, Tugwell P (2011) Standards, guidelines, and norms. J Clin Epidemiol 64: 345-346.

41. Krenzischek D, Wilson L, Newhouse R, Mamaril M, Kane HL (2004) Clinical evaluation of the ASPAN Pain and Comfort Clinical Guideline. J Perianesth Nurs 19: 150-163.

42. Leach MJ, Segal L (2010) Are clinical practical guidelines (CPGs) useful for health services and health workforce planning? A critique of diabetes CPGs. Diabet Med 27: 570-577.

43. Lelgemann M (2009) Kritische Bewertung medizinischer Leitlinien: eine Analyse und Diskussion der Ergebnisse des Deutschen Leitlinien-Clearingverfahrens [PhD thesis]. Cologne: University of Cologne, Faculty of Medicine. Available: http://www.aezq.de/mdb/edocs/pdf/literatur/diss-ml-2009.pdf.

44. Macedo C, Atallah A (2009) Evidence in dentistry guidelines. Sao Paulo Med J 127: 346-349.

45. MacIntyre CR, Plant AJ (1998) Impact of policy and practice on the effectiveness of contact screening for tuberculosis. Prev Med 27: 830-837.

46. Mad P, Geiger Gritsch S, Mittermayr T, Wild C (2009) Tocolysis in preterm labour: a systematic review of evidence-based guidelines, effectiveness and health economic evaluations [German]. Vienna: Ludwig Boltzmann Institut für Health Technology Assessment. Available: http://eprints.hta.lbg.ac.at/825/1/HTA-Projektbericht_Nr30.pdf.

47. Manchikanti L, Datta S, Gupta S, Munglani R, Bryce D, et al. (2010) A critical review of the American Pain Society clinical practice guidelines for interventional techniques: part 2; therapeutic interventions. Pain Physician 13: E215-E264.

48. Matthys J, De Meyere M (2010) Quality evidence important for quality guidelines. CMAJ 182: 1449-1450.

49. McAlister FA, Van Diepen S, Padwal RS, Johnson JA, Majumdar SR (2007) How evidence-based are the recommendations in evidence-based guidelines? PLoS Med 4: 1325-1332.

50. McCormack J, Perry T Jr, Rangno R, Van Breemen C, Wright JM, et al. (2002) Assessing the quality of clinical practice guidelines. Can Med Assoc J 166: 168-169.

51. Muth C, Gensichen J, Beyer M, Hutchinson A, Gerlach FM (2009) The systematic guideline review: method, rationale, and test on chronic heart failure. BMC Health Serv Res 9: 74.

52. Niessen LW, Grijseels E, Koopmanschap M, Rutten F (2007) Economic analysis for clinical practice : the case of 31 national consensus guidelines in the Netherlands. J Eval Clin Pract 13: 68-78.

53. Nitschke M, Smith BJ, Pilotto LS, Pisaniello DL, Abramson MJ, et al. (1999) Respiratory health effects of nitrogen dioxide exposure and current guidelines. Int J Environ Health Res 9: 39-53.

54. Nuckols TK, Wynn BO, Lim Y, Shaw R, Mattke S, et al. (2005) Evaluating medical treatment guideline sets for injured workers in California. Santa Monica: RAND Corporation. Available: http://www.rand.org/content/dam/rand/pubs/monographs/2005/RAND_MG400.pdf.

55. Ollenschläger G (2004) Medical standards and guidelines: definitions and functions [German]. Z Arztl Fortbild Qualitatssich 98: 176-179.

56. Owen A, Kocierz L, Aggarwal N, Hulme J (2010) Comparison of the errors in basic life support performance after training using the 2000 and 2005 ERC guidelines. Resuscitation 81: 766-768.

57. Palmer RH, Banks NJ, Spath P (1995) Checklist for developing guideline derived evaluation instruments. In: Agency for Health Care Policy and Research, editor. Using clinical practice guidelines to evaluate quality of care; volume 2: methods. Rockville: AHCPR. pp. 73-88.

58. Perleth M (1998) Wann ist Verlass auf medizinische Leitlinien? MMW Munch Med Wochenschr 140: 42-46.

59. Persaud N, Mamdani MM (2006) External validity: the neglected dimension in evidence ranking. J Eval Clin Pract 12: 450-453.

60. Roos M, Brodbeck J, Sarkozy A, Battista Chierchia G, De Asmundis C, et al. (2011) A critical analysis of the scientific evidence behind international guidelines related to cardiac arrhythmias. Circ Arrhythm Electrophysiol 4: 202-210.

61. Rowan MS, Carter A (2000) Evaluation of the red blood cell and plasma transfusion guidelines. Int J Qual Health Care 12: 11-17.

62. Rowan MS, Toombs M, Bally G, Walters DJ, Henderson J (1996) Qualitative evaluation of the Canadian Medical Association's counselling guidelines for HIV serologic testing. CMAJ 154: 665-671.

63. Saturno P, Medina F, Valera F, Montilla J, Escolar P, et al. (2003) Validity and reliability of guidelines for neck pain treatment in primary health care: a nationwide empirical analysis in Spain. Int J Qual Health Care 15: 487-493.

64. Scott NA, Moga C, Harstall C (2009) Making the AGREE tool more user-friendly: the feasibility of a user guide based on Boolean operators. J Eval Clin Pract 15: 1061-1073.

65. Shalom E, Shahar Y, Taieb-Maimon M, Bar G, Yarkoni A, et al. (2008) A quantitative assessment of a methodology for collaborative specification and evaluation of clinical guidelines. J Biomed Inform 41: 889-903.

66. Shalom E, Shahar Y, Taieb-Maimon M, Martins S, Vaszar L, et al. (2009) Ability of expert physicians to structure clinical guidelines: reality versus perception. J Eval Clin Pract 15: 1043-1053.

67. Shaneyfelt TM, Centor RM (2009) Reassessment of clinical practice guidelines: go gently into that good night. JAMA 301: 868-869.

68. Shekelle P (2004) The appropriateness method. Med Decis Making 24: 228-231.

69. Shekelle PG, Ortiz E, Rhodes S, Morton SC, Eccles MP, et al. (2001) Validity of the Agency for Healthcare Research and Quality clinical practice guidelines: how quickly do guidelines become outdated? JAMA 286: 1461-1467.

70. Shiffman RN, Karras BT, Agrawal A, Chen R, Marenco L, et al. (2000) GEM: a proposal for a more comprehensive guideline document model using XML. J Am Med Inform Assoc 7: 488-498.

71. Shiffman RN, Michel G (2004) Toward improved guideline quality: using the COGS statement with GEM. Stud Health Technol Inform 107: 159-163.

72. Skonetzki S, Gausepohl HJ, Van der Haak M, Knaebel S, Linderkamp O, et al. (2004) HELEN: a modular framework for representing and implementing clinical practice guidelines. Methods Inf Med 43: 413-426.

73. Snowball R (2005) Critical appraisal of clinical guidelines. In: Dawes M, Davies P, Gray A, editors. Evidence based practice: a primer for health professionals. Edinburgh: Elsevier Churchill Livingstone. pp. 127-131.

74. Spuls PH, Nast A (2010) Evaluation of and perspectives on guidelines: what is important? J Invest Dermatol 130: 2348-2349.

75. Stone J, Austford L, Parker J, Gledhill N, Tremblay G, et al. (2008) AGREEing on Canadian cardiovascular clinical practice guidelines. Can J Cardiol 24: 753-757.

76. Summerfield R, Macduff R, Davis R, Sambrook M, Britton I (2011) Comparative yield of positive brain computed tomography after implementing the NICE or SIGN head injury guidelines in two equivalent urban populations. Clin Radiol 66: 308-314.

77. Trostler N, Myers EF, Snetselaar LG (2008) Determining resting metabolic rate: evaluation of appendix material for ADA evidence-based nutrition practice guidelines using the Dietetics Practice-Based Research Network. Top Clin Nutr 23: 292-305.

78. Van der Steen JT, Ooms ME, Ribbe MW, Van der Wal G (2001) Decisions to treat or not to treat pneumonia in demented psychogeriatric nursing home patients: evaluation of a guideline. Alzheimer Dis Assoc Disord 15: 119-128.

79. Vigna-Taglianti F, Vineis P, Liberati A, Faggiano F (2006) Quality of systematic reviews used in guidelines for oncology practice. Ann Oncol 17: 691-701.

80. Visser VS, De Groot CJM, Luitjes S, Wouters MGAJ, Van Lith J (2011) Comparative analysis of recommendations in local Dutch guidelines on 'hypertension and pregnancy'. Pregnancy Hypertens 1: 176-184.

81. Vlayen J, Aertgeerts B, Hannes K, Sermeus W, Ramaekers D (2005) A systematic review of appraisal tools for clinical practice guidelines: multiple similarities and one common deficit. Int J Qual Health Care 17: 235-242.

82. Von Ferber L, Von Ferber C (2005) To what extent are recommendations of guidelines relevant in everyday practice? An essential question for evaluation studies [German]. Med Klin 100: 340-346.

83. Waring WS, McDonald SH, Good AM, Gordon LD, Bateman DN (2009) Interpretation of clinical guidelines for poisoned patients: positive and negative effects of standard phrases used in TOXBASE. Eur J Clin Pharmacol 65: 1007-1012.

84. Watine J, Bunting P (2008) Mass colorectal cancer screening: methodological quality of practice guidelines is not related to their content validity. Clin Biochem 41: 459-466.

85. Watine J, Friedberg B, Nagy E, Onody R, Oosterhuis W, et al. (2006) Conflict between guideline methodologic quality and recommendation validity: a potential problem for practitioners. Clin Chem 52: 65-72.

86. Weiskopf R (2010) Conflicts of interest in expert-authored practice parameters, standards, guidelines, recommendations. Anesthesiology 113: 751-752.

87. Weiss B (2011) Evidence-based information concerning increasing age in German guideline portals: comparison using heart failure as an example [German]. Z Gerontol Geriatr 44: 85-90.

88. Wens J, Van Royen P (2011) Comment on: "Evaluation and comparison of guidelines for the management of people with type 2 diabetes from eight European countries" by Stone et al. on behalf of the GUIDANCE study group (Diabetes Res Clin Pract 2010, 87(2): 252-60). Diabetes Res Clin Pract 92: 407-408.

89. Wilting I, Egberts A, Heerdink E, Ververs T, Meulenbelt J, et al. (2009) Evaluation of available treatment guidelines for the management of lithium intoxication. Ther Drug Monit 31: 247-260.

90. Wright D, Foster C, Amir Z, Elliott J, Wilson R (2010) Critical appraisal guidelines for assessing the quality and impact of user involvement in research. Health Expect 13: 359-368.

91. Wunderink RG (1998) Clinical practice guidelines for the management of pneumonia: do they work? New Horiz 6: 75-83.

Tool is not for clinical guidelines

1. Appleton JV, Cowley S (1997) Analysing clinical practice guidelines: a method of documentary analysis. J Adv Nurs 25: 1008-1017.

2. Chaves LM, Grypdonck MHF, Defloor T (2010) Protocols for pressure ulcer prevention: are they evidence-based? J Adv Nurs 66: 562-572.

3. Strech D, Schildmann J (2011) Quality of ethical guidelines and ethical content in clinical guidelines: the example of end-of-life decision-making. J Med Ethics 37: 390-396.

4. Veldhuijzen W, Ram PM, Van der Weijden T, Wassink MR, Van der Vleuten CPM (2007) Much variety and little evidence: a description of guidelines for doctor-patient communication. Med Educ 41: 138-145.

Tool published before 1995

1. Cluzeau F, Littlejohns P, Grimshaw JM (1994) Appraising clinical guidelines: towards a "which" guide for purchasers. Qual Health Care 3: 121-122.

2. Field MJ, Lohr KN (1990) Clinical practice guidelines: directions for a new program. Washington: National Academy Press.

3. Field MJ, Lohr KN (1992) Guidelines for clinical practice: from development to use. Washington: National Academy Press.

4. Grilli R, Lomas J (1994) Evaluating the message: the relationship between compliance rate and the subject of a practice guideline. Med Care 32: 202-213.

5. Grimshaw J, Russell I (1993) Achieving health gain through clinical guidelines: I; developing scientifically valid guidelines. Qual Health Care 2: 243-248.

6. Hadorn DC, Baker D (1994) Development of the AHCPR-sponsored heart failure guideline: methodologic and procedural issues. Jt Comm J Qual Improv 20: 539-547.

7. Hayward RSA, Wilson MC, Tunis SR, Bass EB, Rubin HR, et al. (1993) More informative abstracts of articles describing clinical practice guidelines. Ann Intern Med 118: 731-737.

8. Lohr KN, Field MJ (1992) A provisional instrument for assessing clinical practice guidelines. In: Field MJ, Lohr KN, editors. Guidelines for clinical practice: from development to use. Washington: National Academy Press. pp. 346-410.

9. Selker HP (1993) Criteria for adoption in practice of medical practice guidelines. Am J Cardiol 71: 339-341.

Not German or English

1. Apolone G, Bamfi F (1999) A model for an evaluation of clinical guidelines [Italian]. Ricerca e Pratica 15: 116-124.

2. Casi CA, Aizpuru BF, Ibanez PF (2000) Quality analysis of clinical practice guidelines on cardiovascular risks in the Basque country and a proposal for their evaluation [Spanish]. Aten Primaria 26: 585-589.

3. Chen KJ, Jiang YR (2009) Current status and problems in developing clinical guidelines for Chinese medicine and integrative medicine [Chinese]. Zhong Xi Yi Jie He Xue Bao 7: 301-305.

4. Fonfrede M, Couaillac JP, Augereau C, De Mouy D, Lepargneur JP, et al. (2011) Evaluation of the methodological quality of the Remic (microbiology guidelines - bacteriology and mycology) of the Societe Francaise de Microbiologie [French]. Ann Biol Clin (Paris) 69: 239-245.

5. Muscettola G, Rossi A, Scarone S (2010) An appraisal of the major guidelines on the pharmacotherapy of schizophrenia [Italian]. Italian Journal of Psychopathology 16: 196-224.

Published in abstract form only

1. Ahmed A, Soliman S, Awad LA (2010) A validated clinical practice guideline for community health nurses working in tuberculosis out-patient clinics [abstract]. Int J Infect Dis 14: e147.

2. Akhavan P, Schieir O, Hazlewood G, Bykerk V, Bombardier C (2010) Strength of evidence in guidelines regarding the pharmacological management of RA [abstract]. J Rheumatol 2: 1314.

3. Brouwers MC, Kho ME, Makarski J (2010) AGREE II: advancing development, reporting, and evaluation of practice guidelines [abstract]. Cochrane Database Syst Rev (Suppl): 11.

4. Burda BU, Norris S, Holmer HK, Ogden LA, Smith MEB (2010) Quality assessment of clinical practice guidelines for mammography screening in women aged 40-49 years [abstract]. Cochrane Database Syst Rev (Suppl): 91-92.

5. De Seixas MD, Mller U (2009) Systematic review of national and international guidelines on ADHD [abstract]. Eur Psychiatry 24: S395.

6. Gluud C, Greisen G (2004) Comparison of the Cochrane Neonatal Group systematic reviews and Danish guidelines for newborns [abstract]. XII Cochrane Colloquium; 02.-06.04.2004; Ottawa, Canada; [Programme and Abstract Book]: 128.

7. Graham I, Cranney A, Waldegger L, Ooi D, Man Song Hing M, et al. (2002) Systematic review and quality appraisal reveals current osteoporosis guidelines developed with little methodological rigor [abstract]. Annu Meet Int Soc Technol Assess Health Care Int Soc Technol Assess Health Care Meet 18: 115.

8. Hazlewood G, Schieir O, Akhavan P, Bykerk V, Bombardier C (2010) Finding guidelines to AGREE on: a quality appraisal of international guidelines on the pharmacological teatment of rheumatoid arthritis [abstract]. J Rheumatol 2: 1314.

9. Hussain T, Michel G, Shiffman RN (2008) How often is strength of recommendation indicated in guidelines? Analysis of the Yale Guideline Recommendation Corpus [abstract]. AMIA Annu Symp Proc 6: 984.

10. Lalucat-Jo L, Carbonero M, Faus G, Guillamon I, Martnez C, et al. (2009) Comparative study of international clinical practice guidelines about schizophrenia and early psychosis management [abstract]. Eur Arch Psychiatry Clin Neurosci 259: S80-S81.

11. Li-Yu JT, Perez EC, Canete A, Bonifacio L, Llamado LQ, et al. (2010) Consensus guidelines on osteoporosis diagnosis, prevention, and management in the Philippines [abstract]. Osteoporos Int 21: S751.

12. Lo Vecchio A, Giannattasio A, De Masi S, Ortisi MT, Parola L, et al. (2009) Evaluation of quality of guidelines for acute gastroenteritis in children with the AGREE instrument [abstract]. Dig Liver Dis 41: S202.

13. Martinez Zapata MJ, Urrutia G, Gutierrez Ibarluzea I, Moniche F, Ciapponi A, et al. (2006) Agreement between systematic reviews (SRs) and clinical practice guidelines (CPGs) on drug treatment in acute stroke [abstract]. XIV Cochrane Colloquium; 23.-26.10.2006; Dublin, Ireland; programme and abstract book. pp. 116.

14. Muoz-Cano R, Plaza V, Quirce S, Balmes S, Fernandez M, et al. (2010) External evaluation of agreement with the Spanish guidelines for asthma management (GEMA 2009) recommendations performed by an expert panel on asthma [abstract]. Allergy 65: 527-528.

15. Origasa H (2009) Critical appraisal of the Japanese guideline for the management of stroke [abstract]. Value Health 12: A345.

16. Schieir O, Hazlewood G, Akhavan P, Bykerk V, Bombardier C (2010) Adapting ADAPTE: a novel methodology for the development of national clinical practice guidelines [abstract]. J Rheumatol 2: 1315.

17. Schmidt S, Manns MP, Greten TF (2010) Qualitative analysis of international guidelines for diagnosis and treatment of hepatocellular carcinoma by the AGREE instrument (Appraisal of Guidelines for Research and Evaluation) [abstract]. J Hepatol 52: S233.

18. Shalom E, Shahar Y, Taieb-Maimon M, Lunenfeld E (2008) A quality assessment tool for markup-based clinical guidelines [abstract]. AMIA Annu Symp Proc 6: 1127.

19. Shikata S, Sato T, Miyazaki K, Okumura A, Takagaki N, et al. (2010) Description of palliative medicine in guidelines for digestive system cancer medical care [abstract]. J Clin Oncol 28: e19628.

20. Siering U, Stich AK, Kirchner H (2006) Do guidelines reflect current best evidence? A comparison of guideline recommendations on rapid-acting insulin analogues and conclusions of a systematic review [abstract]. XIV Cochrane Colloquium; 23.-26.10.2006; Dublin, Ireland; programme and abstract book. pp. 101.

21. Simmons CE, Hogeveen S, Nichols J, Trudeau-Tavara S, Quan M (2010) Quality and consistency in breast cancer clinical guidelines internationally: are we globally consistent? [abstract]. J Clin Oncol 28: 6096.

22. Stone MA, Wilkinson JC, Charpentier G, Clochard N, Lindblad U, et al. (2009) Appraisal and comparison of guidelines for the management of people with type 2 diabetes in eight European countries [abstract]. Diabetologia 52: S101.

23. Vlayen J, Aertgeerts B, Hannes K, Sermeus W, Ramaekers D (2006) A systematic review of appraisal tools for clinical practice guidelines: multiple similarities and one common deficit [abstract]. XIV Cochrane Colloquium; 23.-26.10.2006; Dublin, Ireland; programme and abstract book. pp. 101.

24. Weinmann S (2009) European and international guidelines on schizophrenia: consistencies and disagreements [abstract]. Eur Arch Psychiatry Clin Neurosci 259: S20-S21.

Letter to the editor

1. Burls A (2010) AGREE II: improving the quality of clinical care. Lancet 376: 1128-1129.

Not available from local libraries, interlibrary loan, or author request

1. Cluzeau F, Littlejohns P, Grimshaw J, Feder G (1997) Appraisal instrument for clinical guidelines. London: St George’s Hospital Medical School.

2. Littlejohns P, Cluzeau F (1997) Promoting the rigorous development of clinical guidelines in Europe through the creation of a common appraisal instrument. Amsterdam: Scientific Basis for Health Services.

3. Moja P, Lari C, Trisolini R, Auxilia F (2003) Analysis of some variables concerning guidelines adopted in a hospital in Milan: quantity, quality and physicians' attitude; which is the reciprocal influence of these factors? Eur J Public Health 13: 146-151.

4. Savoie J, Green CJ, Bassett K, Kazanjian A (1996) Critical appraisal criteria for clinical practice guidelines. Vancouver: British Columbia Office of Health Technology Assessment.

5. Scottish Intercollegiate Guidelines Network (1995) Clinical guidelines: criteria for appraisal for national use. Edinburgh: SIGN.

Multiple publication of the tool

1. Brok J, Greisen G, Madsen LP, Tilma K, Faerk J, et al. (2008) Agreement between Cochrane Neonatal reviews and clinical practice guidelines for newborns in Denmark: a cross-sectional study. Arch Dis Child Fetal Neonatal Ed 93: F225-F229.

2. Brouwers M, Kho ME, Browman G, Burgers J, Cluzeau F, et al. (2010) AGREE II: advancing guideline development, reporting and evaluation in health care. J Clin Epidemiol 63: 1308-1311.

3. Brouwers M, Kho ME, Browman G, Burgers JS, Cluzeau F, et al. (2010) AGREE II: advancing guideline development, reporting, and evaluation in health care. Prev Med 51: 421-424.

4. Nuckols TK, Lim YW, Wynn BO, Mattke S, MacLean C, et al. (2008) Rigorous development does not ensure that guidelines are acceptable to a panel of knowledgeable providers. J Gen Intern Med 23: 37-44.

5. Ollenschläger G, Helou A, Kostovic-Cilic L, Perleth M, Raspe HH, et al. (1998) Checklist for methodological quality of guidelines: a contribution to quality promotion of medical guidelines [German]. Z Arztl Fortbild Qualitatssich 92: 191-194.

6. Oxman AD, Schünemann HJ, Fretheim A (2006) Improving the use of research evidence in guideline development: 16; evaluation. Health Res Policy Syst 4: 28.

Old version of the tool

1. ADAPTE Collaboration (2007) Resource toolkit for guideline adaptation: version 1.0. Available: http://www.adapte.org/www/upload/actualite/pdf/Manual%20&%20Toolkit.pdf. Accessed 24 June 2008.

2. AGREE Collaboration (2001) Appraisal of Guidelines For Research & Evaluation: AGREE instrument. London: St George’s Hospital Medical School.

3. AGREE Collaboration (2003) Appraisal of Guidelines For Research & Evaluation: AGREE instrument; training manual. AGREE Collaboration.

4. Ärztliche Zentralstelle Qualitätssicherung (2000) Checkliste Methodische Qualität von Leitlinien: 2. Version (8/1999); gültig bis 31. August 2000; Bewertungsinstrument des Leitlinien-Clearingverfahrens. Dtsch Arztebl 97: A1170-A1172.

5. Ärztliche Zentralstelle Qualitätssicherung, Bundesärztekammer, Kassenärztliche Bundesvereinigung (1998) Checkliste "Methodische Qualität von Leitlinien": 1. Version; Gültigkeit: 1.2.1998 bis 31.1.1999. Dtsch Arztebl 95: A2576-A2578.

6. Helou A, Kostovic-Clic L, Ollenschläger G (1998) Nutzermanual zur Checkliste "Methodische Qualität von Leitlinien". Available: http://www.leitlinien.de/mdb/edocs/pdf/info/nutzermanual-check-ll.pdf. Accessed 18 November 2011.

7. Kopp I, Lelgemann M (2005) The AGREE (Appraisal of Guidelines Research and Evaluation) instrument [German]. Z Arztl Fortbild Qualitatssich 99: 497-498, 469-470.

Application of an already identified appraisal tool

1. Aass Y, McConnell H, Perrier L, Woodbury MG, Sibbald RG (2009) Process for developing evidence-informed practice recommendations: venous leg ulcer example. Adv Skin Wound Care 22: 133-140.

2. Ackman ML, Druteika D, Tsuyuki RT (2000) Levels of evidence in cardiovascular clinical practice guidelines. Can J Cardiol 16: 1249-1254.

3. Alonso-Coello P, Irfan A, Sola I, Gich I, Delgado-Noguera M, et al. (2010) The quality of clinical practice guidelines over the last two decades: a systematic review of guideline appraisal studies. Qual Saf Health Care 19: e58.

4. Appleyard TL, Mann CH, Khan KS (2006) Guidelines for the management of pelvic pain associated with endometriosis: a systematic appraisal of their quality. BJOG 113: 749-757.

5. Arnau JM, Vallano A, Lopez A, Pellise F, Delgado MJ, et al. (2006) A critical review of guidelines for low back pain treatment. Eur Spine J 15: 543-553.

6. Aus G, Chapple C, Hanus T, Irani J, Lobel B, et al. (2009) The European Association of Urology (EAU) guidelines methodology: a critical evaluation. Eur Urol 56: 859-864.

7. Baird A, Olarinde O, Talbot M (2007) Evaluation, using two assessment instruments, of the American and British national guidelines for the management of sexually transmissible and genital infections. Sex Health 4: 255-260.

8. Barajas-Nava L, Sola I, Delgado-Noguera M, Gich I, Villagran C, et al. (2010) Quality assessment of clinical practice guidelines in perioperative care: a systematic appraisal. Qual Saf Health Care 19: e50.

9. Berner MM, Habbig S, Harter M (2004) Quality of guidelines for the treatment of alcohol related disorders: a systematic review and content analysis [German]. Fortschr Neurol Psychiatr 72: 696-704.

10. Berti E, Grilli R (2003) Practice guidelines and clinical governance: do the means match with the ends? A quality appraisal of local practice guidelines. Clinical Governance 8: 312-317.

11. Boluyt N, Lincke CR, Offringa M (2005) Quality of evidence-based pediatric guidelines. Pediatrics 115: 1378-1391.

12. Bouwmeester W, Van Enst A, Van Tulder MW (2009) Quality of low back pain guidelines improved. Spine 34: 2562-2567.

13. Brosseau L, Graham I, Casimiro L, MacLeay L, Cleaver S, et al. (2004) What is the quality of clinical practice guidelines accessible on the world wide web for the treatment of musculoskeletal conditions in physiotherapy? Physiother Theory Pract 20: 91-105.

14. Brouwers MC, Rawski E, Spithoff K, Oliver TK (2011) Inventory of Cancer Guidelines: a tool to advance the guideline enterprise and improve the uptake of evidence. Expert Rev Pharmacoecon Outcomes Res 11: 151-161.

15. Brozek J, Jankowski M, Placzkiewicz-Jankowska E, Jaeschke R (2009) International Diabetes Federation document concerning postmeal glycemic control: assessment of quality of clinical practice guidelines using AGREE instrument. Pol Arch Med Wewn 119: 18-24.

16. Burda BU, Norris SL, Holmer HK, Ogden LA, Smith ME (2011) Quality varies across clinical practice guidelines for mammography screening in women aged 40-49 years as assessed by AGREE and AMSTAR instruments. J Clin Epidemiol 64: 968-976.

17. Burgers JS, Cluzeau FA, Hanna SE, Hunt C, Grol R (2003) Characteristics of high-quality guidelines: evaluation of 86 clinical guidelines developed in ten European countries and Canada. Int J Technol Assess Health Care 19: 148-157.

18. Burgers JS, Fervers B, Haugh M, Brouwers M, Browman G, et al. (2004) International assessment of the quality of clinical practice guidelines in oncology using the Appraisal of Guidelines and Research and Evaluation Instrument. J Clin Oncol 22: 2000-2007.

19. Cates JR, Young DN, Bowerman DS, Porter RC (2006) An independent AGREE evaluation of the Occupational Medicine Practice Guidelines. Spine J 6: 72-77.

20. Cates JR, Young DN, Guerriero DJ, Jahn WT, Armine JP, et al. (2001) Evaluating the quality of clinical practice guidelines. J Manipulative Physiol Ther 24: 170-176.

21. Cates JR, Young DN, Guerriero DJ, Jahn WT, Armine JP, et al. (2003) An independent assessment of chiropractic practice guidelines. J Manipulative Physiol Ther 26: 282-286.

22. Coomarasamy A, Ola B, Gee H, Khan KS (2003) Quality of brief guidelines produced by professional bodies: a study of the 'green-top' guidelines by the UK Royal College of Obstetricians and Gynaecologists. J Obstet Gynaecol 23: 479-483.

23. Cranney A, Waldegger L, Graham ID, Man-Son-Hing M, Byszewski A, et al. (2002) Systematic assessment of the quality of osteoporosis guidelines. BMC Musculoskelet Disord 3: 20.

24. Cruse H, Winiarek M, Marshburn J, Clark O, Djulbegovic B (2002) Quality and methods of developing practice guidelines. BMC Health Serv Res 2: 1.

25. Dagenais S, Tricco A, Haldeman S (2010) Synthesis of recommendations for the assessment and management of low back pain from recent clinical practice guidelines. Spine J 10: 514-529.

26. De Boer WE, Bruinvels DJ, Rijkenberg AM, Donceel P, Anema JR (2009) Evidence-based guidelines in the evaluation of work disability: an international survey and a comparison of quality of development. BMC Public Health 9: 349.

27. De Haas ERM, De Vijlder HC, Van Reesema W, Van Everdingen JJE, Neumann HAM (2007) Quality of clinical practice guidelines in dermatological oncology. J Eur Acad Dermatol Venereol 21: 1193-1198.

28. Debourdeau P, Beckers M, Gerome P, Durant C, Lacoin Q, et al. (2011) How to improve the implementation of guidelines on cancer-related thrombosis. Expert Rev Anticancer Ther 11: 473-483.

29. Delgado-Noguera M, Tort S, Bonfill X, Gich I, Alonso-Coello P (2009) Quality assessment of clinical practice guidelines for the prevention and treatment of childhood overweight and obesity. Eur J Pediatr 168: 789-799.

30. Devroey D, Vantomme K, Betz W, Vandevoorde J, Kartounian J (2004) A review of the treatment guidelines on the management of low levels of high-density lipoprotein cholesterol. Cardiology 102: 61-66.

31. Diez F (2002) Guidelines for the diagnosis of osteoporosis by densitometric methods. J Manipulative Physiol Ther 25: 403-415.

32. Esandi ME, Ortiz Z, Chapman E, Dieguez MG, Mejia R, et al. (2008) Production and quality of clinical practice guidelines in Argentina (1994-2004): a cross-sectional study. Implement Sci 3: 43.

33. Faggion C Jr (2008) Clinician assessment of guidelines that support common dental procedures. J Evid Based Dent Pract 8: 1-7.

34. Ferket BS, Colkesen EB, Visser JJ, Spronk S, Kraaijenhagen RA, et al. (2010) Systematic review of guidelines on cardiovascular risk assessment: which recommendations should clinicians follow for a cardiovascular health check? Arch Intern Med 170: 27-40.

35. Ferket BS, Genders TSS, Colkesen EB, Visser JJ, Spronk S, et al. (2011) Systematic review of guidelines on imaging of asymptomatic coronary artery disease. J Am Coll Cardiol 57: 1591-1600.

36. Ferket BS, Grootenboer N, Colkesen EB, Visser JJ, Van Sambeek MR, et al. (2012) Systematic review of guidelines on abdominal aortic aneurysm screening. J Vasc Surg 55: 1296-1304.

37. Fervers B, Burgers JS, Haugh MC, Brouwers M, Browman G, et al. (2005) Predictors of high quality clinical practice guidelines: examples in oncology. Int J Qual Health Care 17: 123-132.

38. Foureur M, Ryan CL, Nicholl M, Homer C (2010) Inconsistent evidence: analysis of six national guidelines for vaginal birth after cesarean section. Birth 37: 3-10.

39. Franzen D, Fessler J, Fischer J, Geraedts M, Graf HJ, et al. (2004) The guideline clearing project COPD: recommendations for a national guideline [German]. Pneumologie 58: 858-862.

40. Freel A, Shiloach M, Weigelt J, Beilman G, Mayberry J, et al. (2008) American College of Surgeons Guidelines Program: a process for using existing guidelines to generate best practice recommendations for central venous access. J Am Coll Surg 207: 676-682.

41. Gaebel W, Weinmann S, Sartorius N, Rutz W, McIntyre JS (2005) Schizophrenia practice guidelines: international survey and comparison. Br J Psychiatry 187: 248-255.

42. Gallardo CR, Rigau D, Irfan A, Ferrer A, Cayla JA, et al. (2010) Quality of tuberculosis guidelines: urgent need for improvement. Int J Tuberc Lung Dis 14: 1045-1051.

43. Geusens PP, Lems WF, Verhaar HJJ, Leusink G, Goemaere S, et al. (2006) Review and evaluation of the Dutch guidelines for osteoporosis. J Eval Clin Pract 12: 539-548.

44. Glenny AM, Worthington H, Clarkson J, Esposito M (2009) The appraisal of clinical guidelines in dentistry. Eur J Oral Implantol 2: 135-143.

45. Goergen S, Rumbold G, Compton G, Harris C (2010) Systematic review of current guidelines, and their evidence base, on risk of lactic acidosis after administration of contrast medium for patients receiving metformin. Radiology 254: 261-269.

46. Gorman SK, Chung MH, Slavik RS, Zed P, Wilbur K, et al. (2010) A critical appraisal of the quality of critical care pharmacotherapy clinical practice guidelines and their strength of recommendations. Intensive Care Med 36: 1636-1643.

47. Graham ID, Beardall S, Carter AO, Glennie J, Hebert PC, et al. (2001) What is the quality of drug therapy clinical practice guidelines in Canada? CMAJ 165: 157-163.

48. Grimshaw GM, Khunti K, Baker R (2001) Diagnosis of heart failure in primary care: an assessment of international guidelines. Br J Gen Pract 51: 384-386.

49. Harpole LH, Kelley MJ, Schreiber G, Toloza EM, Kolimaga J, et al. (2003) Assessment of the scope and quality of clinical practice guidelines in lung cancer. Chest 123(1 Suppl): 7S-20S.

50. Helou A, Perleth M, Bitzer EM, Dorning H, Schwartz FW (1998) Methodological quality of clinical practice guidelines in Germany: results of a systemic assessment of guidelines presented on the Internet [German]. Z Arztl Fortbild Qualitatssich 92: 421-428.

51. Holtmann I, Luhmann D, Raspe H (2000) International practice guidelines on the management of osteoporosis: assessment of methodologic quality [German]. Z Arztl Fortbild Qualitatssich 94: 483-490.

52. Hulshof C, Hoenen J (2007) Evidence-based practice guidelines in OHS: are they agree-able? Ind Health 45: 26-31.

53. Hurdowar A, Graham I, Bayley M, Harrison M, Wood-Dauphinee S, et al. (2007) Quality of stroke rehabilitation clinical practice guidelines. J Eval Clin Pract 13: 657-664.

54. Iorio A, Basileo M, Marchesini E, Materazzi M, Marchesi M, et al. (2008) The good use of plasma: a critical analysis of five international guidelines. Blood Transfus 6: 18-24.

55. Irani J, Brown CT, Van der Meulen J, Emberton M (2003) A review of guidelines on benign prostatic hyperplasia and lower urinary tract symptoms: are all guidelines the same? BJU Int 92: 937-942.

56. Irani S, Rashidian A, Yousefi-Nooraie R, Soltani A (2011) Evaluating clinical practice guidelines developed for the management of thyroid nodules and thyroid cancers and assessing the reliability and validity of the AGREE instrument. J Eval Clin Pract 17: 729-736.

57. Kinnunen-Amoroso M, Pasternack I, Mattila S, Parantainen A (2009) Evaluation of the practice guidelines of Finnish Institute of Occupational Health with AGREE instrument. Ind Health 47: 689-693.

58. Kis E, Szegesdi I, Dobos E, Nagy E, Boda K, et al. (2010) Quality assessment of clinical practice guidelines for adaptation in burn injury. Burns 36: 606-615.

59. Lacasse Y, Ferreira I, Brooks D, Newman T, Goldstein RS (2001) Critical appraisal of clinical practice guidelines targeting chronic obstructive pulmonary disease. Arch Intern Med 161: 69-74.

60. Lepanto L, Tang A, Murphy-Lavallee J, Billiard JS (2011) The Canadian Association of Radiologists Guidelines for the prevention of contrast-induced nephropathy: a critical appraisal. Can Assoc Radiol J 62: 238-242.

61. Lindena G, Diener HC, Hildebrandt J, Klinger R, Maier C, et al. (2002) Guidelines in pain treatment: methodical quality of guidelines for treatment of pain patients [German]. Schmerz 16: 194-204.

62. Littlejohns P, Cluzeau F, Bale R, Grimshaw J, Feder G, et al. (1999) The quantity and quality of clinical practice guidelines for the management of depression in primary care in the UK. Br J Gen Pract 49: 205-210.

63. Lo Vecchio A, Giannattasio A, Duggan C, De Masi S, Ortisi M, et al. (2010) Evaluation of the quality of guidelines for acute gastroenteritis in children, with the AGREE instrument [abstract]. J Pediatr Gastroenterol Nutr 50: E27.

64. Lo Vecchio A, Giannattasio A, Duggan C, De Masi S, Ortisi MT, et al. (2011) Evaluation of the quality of guidelines for acute gastroenteritis in children with the AGREE instrument. J Pediatr Gastroenterol Nutr 52: 183-189.

65. Lopez-Olivo M, Kallen M, Ortiz Z, Skidmore B, Suarez-Almazor M (2008) Quality appraisal of clinical practice guidelines and consensus statements on the use of biologic agents in rheumatoid arthritis: a systematic review. Arthritis Rheum 59: 1625-1638.

66. Loveday B, Srinivasa S, Vather R, Mittal A, Petrov M, et al. (2010) High quantity and variable quality of guidelines for acute pancreatitis: a systematic review. Am J Gastroenterol 105: 1466-1476.

67. Lyman G, Kleiner J (2011) Summary and comparison of myeloid growth factor guidelines in patients receiving cancer chemotherapy. Cancer Treat Res 157: 145-165.

68. Lyman GH, Kleiner JM (2007) Summary and comparison of myeloid growth factor guidelines in patients receiving cancer chemotherapy. J Natl Compr Canc Netw 5: 217-228.

69. MacDermid JC (2004) The quality of clinical practice guidelines in hand therapy. J Hand Ther 17: 200-209.

70. MacLeod FE, Harrison MB, Graham ID (2002) The process of developing best practice guidelines for nurses in Ontario: risk assessment and prevention of pressure ulcers. Ostomy Wound Manage 48: 30-32, 34-38.

71. Mahmud M, Mazza D (2010) Preconception care of women with diabetes: a review of current guideline recommendations. BMC Womens Health 10: 5.

72. Manchikanti L, Singh V, Derby R, Schultz D, Benyamin R, et al. (2008) Reassessment of evidence synthesis of occupational medicine practice guidelines for interventional pain management. Pain Physician 11: 393-482.

73. McDonagh R, White C, Singh G, Mohide P (2002) The quality of obstetrical clinical practice guidelines promulgated by a specialty society. J Obstet Gynaecol Can 24: 957-962.

74. McIntosh HM, Neal RD, Rose P, Watson E, Wilkinson C, et al. (2009) Follow-up care for men with prostate cancer and the role of primary care: a systematic review of international guidelines. Br J Cancer 100: 1852-1860.

75. McKinley S, Elliott R (2008) Implications for Australian practice of North American guidelines for the support of the family in patient-centred intensive care. Collegian 15: 11-17.

76. McNair R, Hegarty K (2010) Guidelines for the primary care of lesbian, gay, and bisexual people: a systematic review. Ann Fam Med 8: 533-541.

77. Minhas R (2007) Eminence-based guidelines: a quality assessment of the second Joint British Societies' guidelines on the prevention of cardiovascular disease. Int J Clin Pract 61: 1137-1144.

78. Misso M, Pitt V, Jones K, Barnes H, Piterman L, et al. (2008) Quality and consistency of clinical practice guidelines for diagnosis and management of osteoarthritis of the hip and knee: a descriptive overview of published guidelines. Med J Aust 189: 394-399.

79. Murphy AYMT, Van Teijlingen ER, Gobbi MO (2006) Inconsistent grading of evidence across countries: a review of low back pain guidelines. J Manipulative Physiol Ther 29: 576-581, 581.e1-581.e2.

80. Nast A, Spuls PH, Ormerod AD, Reytan N, Saiag PH, et al. (2009) A critical appraisal of evidence-based guidelines for the treatment of psoriasis vulgaris: 'AGREE-ing' on a common base for European evidence-based psoriasis treatment guidelines. J Eur Acad Dermatol Venereol 23: 782-787.

81. Navarro Puerto MA, Ibarluzea I, Ruiz OG, Alvarez FM, Herreros RG, et al. (2008) Analysis of the quality of clinical practice guidelines on established ischemic stroke. Int J Technol Assess Health Care 24: 333-341.

82. Nelen WLDM, Van der Pluijm RW, Hermens RPMG, Bergh C, De Sutter P, et al. (2008) The methodological quality of clinical guidelines of the European Society of Human Reproduction and Embryology (ESHRE). Hum Reprod 23: 1786-1792.

83. Novara G, Galfano A, Gardi M, Ficarra V, Boccon-Gibod L, et al. (2006) Critical review of guidelines for BPH diagnosis and treatment strategy. European Urology Supplements 5: 418-429.

84. Otten K, Geraedts M, Kugler C (2006) Critical appraisal of stroke guidelines [German]. Z Arztl Fortbild Qualitatssich 100: 275-281.

85. Ou Y, Goldberg I, Migdal C, Lee PP (2011) A critical appraisal and comparison of the quality and recommendations of glaucoma clinical practice guidelines. Ophthalmology 118: 1017-1023.

86. Peloso PM, Carroll LJ, Cassidy JD, Borg J, Von Holst H, et al. (2004) Critical evaluation of the existing guidelines on mild traumatic brain injury. J Rehabil Med 36(Suppl 43): 106-112.

87. Pencharz JN, Grigoriadis E, Jansz GF, Bombardier C (2002) A critical appraisal of clinical practice guidelines for the treatment of lower-limb osteoarthritis. Arthritis Res 4: 36-44.

88. Poitras S, Avouac J, Rossignol M, Avouac B, Cedraschi C, et al. (2007) A critical appraisal of guidelines for the management of knee osteoarthritis using Appraisal of Guidelines Research and Evaluation criteria. Arthritis Res Ther 9: R126.

89. Potting C, Mistiaen P, Poot E, Blijlevens N, Donnelly P, et al. (2009) A review of quality assessment of the methodology used in guidelines and systematic reviews on oral mucositis. J Clin Nurs 18: 3-12.

90. Rosumeck S, Sporbeck B, Rzany B, Nast A (2011) Disclosure of potential conflicts of interest in dermatological guidelines in Germany: an analysis; status quo and quo vadis [German]. J Dtsch Dermatol Ges 9: 297-304.

91. Rowe R (2010) Local guidelines for the transfer of women from midwifery unit to obstetric unit during labour in England: a systematic appraisal of their quality. Qual Saf Health Care 19: 90-94.

92. Rusnak M, Mauritz W, Lecky F, Kaniansky M, Brazinova A (2008) Evaluation of traumatic brain injury guidelines using AGREE instrument. Bratisl Lek Listy 109: 374-380.

93. Shimbo T, Fukui T, Ishioka C, Okamoto K, Okamoto T, et al. (2010) Quality of guideline development assessed by the Evaluation Committee of the Japan Society of Clinical Oncology. Int J Clin Oncol 15: 227-233.

94. Sica G, Harker-Murray P, Montori VM, Smith SA (2002) Adherence of published diabetes mellitus practice guidelines to methodologic standards of guideline development. Endocrinol Metab Clin North Am 31: 819-828.

95. Sinuff T, Patel R, Adhikari N, Meade M, Schünemann HJ, et al. (2008) Quality of professional society guidelines and consensus conference statements in critical care. Crit Care Med 36: 1049-1058.

96. Spence K, Henderson-Smart D, New K, Evans C, Whitelaw J, et al. (2010) Evidenced-based clinical practice guideline for management of newborn pain. J Paediatr Child Health 46: 184-192.

97. Staal JB, Hlobil H, Van Tulder MW, Waddell G, Burton AK, et al. (2003) Occupational health guidelines for the management of low back pain: an international comparison. Occup Environ Med 60: 618-626.

98. Stanback J, Katz K (2002) Methodological quality of WHO medical eligibility criteria for contraceptive use. Contraception 66: 1-5.

99. Stein DJ, Ipser J, McAnda N (2009) Pharmacotherapy of posttraumatic stress disorder: a review of meta-analyses and treatment guidelines. CNS Spectr 14: 25-31.

100. Steurer J (1998) Critical evaluation of "guidelines" [German]. Praxis 87: 199-204.

101. Stiegler M, Rummel C, Wahlbeck K, Kissling W, Leucht S (2005) European psychiatric treatment guidelines: is the glass half full or half empty? Eur Psychiatry 20: 554-558.

102. Stone MA, Wilkinson JC, Charpentier G, Clochard N, Grassi G, et al. (2010) Evaluation and comparison of guidelines for the management of people with type 2 diabetes from eight European countries. Diabetes Res Clin Pract 87: 252-260.

103. Szegesdi I, Kis E, Zimnyi M, Vimlti L, Kemeny L (2009) Assessment of the scope and quality of clinical practice guidelines in burn injury [abstract]. Eur J Anaesthesiol 26: 16.

104. Tan J, Wolfe B, Bulatovic R, Jones E, Lo A (2010) Critical appraisal of quality of clinical practice guidelines for treatment of psoriasis vulgaris, 2006-2009. J Invest Dermatol 130: 2389-2395.

105. Thole H, Weingart O, Lampert U, Bassler D, Fessler J, et al. (2003) The German guideline clearing-project on bronchial asthma: part 1; methodology and results of formal appraisal [German]. Pneumologie 57: 459-467.

106. Thomeczek C, Lampert U, Brune K, Hasenbring M, Kramer J, et al. (2003) Acute backache guideline clearing process: methodology and results of formal evaluation [German]. Z Orthop Ihre Grenzgeb 141: 11-17.

107. Turner TJ, Barnes H, Reid J, Garrubba M (2010) Evidence for perinatal and child health care guidelines in crisis settings: can Cochrane help? BMC Public Health 10: 170.

108. Van de Velde S, Heselmans A, Donceel P, Vandekerckhove P, Ramaekers D, et al. (2011) Rigour of development does not AGREE with recommendations in practice guidelines on the use of ice for acute ankle sprains. BMJ Qual Saf 20: 747-755.

109. Van der Wees PJ, Hendriks EJM, Custers JWH, Burgers JS, Dekker J, et al. (2007) Comparison of international guideline programs to evaluate and update the Dutch program for clinical guideline development in physical therapy. BMC Health Serv Res 7: 191.

110. Van Diermen DE, Aartman IHA, Baart JA, Hoogstraten J, Van der Waal I (2009) Dental management of patients using antithrombotic drugs: critical appraisal of existing guidelines. Oral Surg Oral Med Oral Pathol Oral Radiol Endod 107: 616-624.

111. Van Tulder MW, Tuut M, Pennick V, Bombardier C, Assendelft WJJ (2004) Quality of primary care guidelines for acute low back pain. Spine 29: E357-E362.

112. Vasse E, Vernooij-Dassen M, Cantegreil I, Franco M, Dorenlot P, et al. (2012) Guidelines for psychosocial interventions in dementia care: a European survey and comparison. Int J Geriatr Psychiatry 27: 40-48.

113. Verwey B, Van Waarde JA, Van Rooij IALM, Gerritsen G, Zitman FG (2006) Availability, content and quality of local guidelines for the assessment of suicide attempters in university and general hospitals in the Netherlands. Gen Hosp Psychiatry 28: 336-342.

114. Vitry A, Zhang Y (2008) Quality of Australian clinical guidelines and relevance to the care of older people with multiple comorbid conditions. Med J Aust 189: 360-365.

115. Warner JP, Blizard R (1998) How to appraise clinical guidelines. Psychiatr Bull R Coll Psychiatr 22: 759-761.

116. Wegman A, Van der Windt D, Van Tulder MW, Stalman W, De Vries T (2004) Nonsteroidal antiinflammatory drugs or acetaminophen for osteoarthritis of the hip or knee? A systematic review of evidence and guidelines. J Rheumatol 31: 344-354.

117. Weingart O, Sonntag D, Trapp H, Bartsch HH, Baumeister R, et al. (2004) The German Guideline Clearinghouse on breast cancer: the need for frequent updating of breast cancer guidelines requires effective guideline updating procedures [German]. Z Arztl Fortbild Qualitatssich 98: 403-411.

118. Wens J, Dirven K, Mathieu C, Paulus D, Van Royen P (2007) Quality indicators for type-2 diabetes care in practice guidelines: an example from six European countries. Prim Care Diabetes 1: 17-23.

119. Wimpenny P, Van Zelm R (2007) Appraising and comparing pressure ulcer guidelines. Worldviews Evid Based Nurs 4: 40-50.

120. Zadvinskis I, Grudell B (2010) Clinical practice guideline appraisal using the AGREE instrument: renal screening. Clin Nurse Spec 24: 209-214.

121. Zhang W, Doherty M (2006) EULAR recommendations for knee and hip osteoarthritis: a critique of the methodology. Br J Sports Med 40: 664-669.

Methods for guideline development without criteria for guideline appraisal

1. Agrawal A, Shiffman RN (2001) Evaluation of guideline quality using GEM-Q. Stud Health Technol Inform 84: 1097-1101.

2. AGREE Collaboration (2003) Development and validation of an international appraisal instrument for assessing the quality of clinical practice guidelines: the AGREE project. Qual Saf Health Care 12: 18-23.

3. Albert US, Kopp I (2010) What are S1-, S2- and S3-guidelines? [German]. Gynakol Prax 34: 3-9.

4. Anagnostopoulos C, Harbinson M, Kelion A, Kundley K, Loong CY, et al. (2003) Procedure guidelines for radionuclide myocardial perfusion imaging. Nucl Med Commun 24: 1105-1119.

5. Anders RL, Tomai JS, Clute RM, Olson T (1997) Development of a scientifically valid coordinated care path. J Nurs Adm 27: 45-52.

6. Appraisal of Guidelines, Research, and Evaluation in Europe (AGREE) Collaborative Group (2000) Guideline development in Europe: an international comparison. Int J Technol Assess Health Care 16: 1039-1049.

7. Arbeitsgemeinschaft der Wissenschaftlichen Medizinischen Fachgesellschaften, Ärztliche Zentralstelle Qualitätssicherung (2001) The Guideline Manual: development and implementation of guidelines in medicine [German]. Z Arztl Fortbild Qualitatssich 95(Suppl 1): 1-84.

8. Arispe IE, Bernstein JF (1996) Linking evaluation and program development: the case of AHCPR-supported clinical practice guidelines. Eval Health Prof 19: 377-393.

9. Aust B, Ohmann C (2000) Previous experiences with the evaluation of guidelines: disillusionment after an enthusiastic start [German]. Z Arztl Fortbild Qualitatssich 94: 365-371.

10. Aust B, Ohmann C (2000) Previous experiences with evaluation of practice guidelines [German]. Zentralbl Chir 125(Suppl 2): 141-145.

11. Balshem H, Helfand M, Schünemann HJ, Oxman AD, Kunz R, et al. (2011) GRADE guidelines: 3; rating the quality of evidence. J Clin Epidemiol 64: 401-406.

12. Baltas CS, Balanika AP, Raptou PD, Tournis S, Lyritis GP (2005) Clinical practice guidelines proposed by the Hellenic Foundation of Osteoporosis for the management of osteoporosis based on DXA results. J Musculoskelet Neuronal Interact 5: 388-392.

13. Basinski AS (1995) Evaluation of clinical practice guidelines. CMAJ 153: 1575-1581.

14. Beyer M, Scherer M, Wollny A, Chenot JF, Baum E, et al. (2010) Redesigning the guideline development concept ("Zehnstufenplan") of the German College of General Practitioners and Family Physicians [German]. Z Allgemeinmed 86: 19-25.

15. Blackwell J (2004) Evaluation and treatment of hyperthyroidism and hypothyroidism. J Am Acad Nurse Pract 16: 422-425.

16. Bollini P, Pampallona S, Nieddu S, Bianco M, Tibaldi G, et al. (2008) Indicators of conformance with guidelines of schizophrenia treatment in mental health services. Psychiatr Serv 59: 782-791.

17. Brouwers MC, Kho ME, Browman GP, Burgers JS, Cluzeau F, et al. (2010) AGREE II: advancing guideline development, reporting and evaluation in health care. CMAJ 182: E839-E842.

18. Brouwers MC, Kho ME, Browman GP, Burgers JS, Cluzeau F, et al. (2010) Development of the AGREE II, part 2: assessment of validity of items and tools to support application. CMAJ 182: E472-E478.

19. Brouwers MC, Kho ME, Browman GP, Burgers JS, Cluzeau F, et al. (2010) Development of the AGREE II, part 1: performance, usefulness and areas for improvement. CMAJ 182: 1045-1052.

20. Buchan HA, Currie KC, Lourey EJ, Duggan GR (2010) Australian clinical practice guidelines: a national study. Med J Aust 192: 490-494.

21. Burgers JS, Grol R, Klazinga NS, Makela M, Zaat J (2003) Towards evidence-based clinical practice: an international survey of 18 clinical guideline programs. Int J Qual Health Care 15: 31-45.

22. Burgers JS, Grol R, Klazinga NS, Van der Bij AK, Makela M, et al. (2003) International comparison of 19 clinical guideline programs: a survey of the AGREE Collaboration [German]. Z Arztl Fortbild Qualitatssich 97: 81-88.

23. Bussieres AE, Peterson C, Taylor JAM (2007) Diagnostic imaging practice guidelines for musculoskeletal complaints in adults: an evidence-based approach; introduction. J Manipulative Physiol Ther 30: 617-683.

24. Bussieres AE, Peterson C, Taylor JAM (2008) Diagnostic imaging guideline for musculoskeletal complaints in adults: an evidence-based approach; part 2; upper extremity disorders. J Manipulative Physiol Ther 31: 2-32.

25. Bussieres AE, Taylor JAM, Peterson C (2007) Diagnostic imaging practice guidelines for musculoskeletal complaints in adults: an evidence-based approach; part 1; lower extremity disorders. J Manipulative Physiol Ther 30: 684-717.

26. Bussieres AE, Taylor JAM, Peterson C (2008) Diagnostic imaging practice guidelines for musculoskeletal complaints in adults: an evidence-based approach; part 3; spinal disorders. J Manipulative Physiol Ther 31: 33-88.

27. Carneiro AV (2004) Methodological appraisal of guidelines: the AGREE instrument. Rev Port Cardiol 23: 447-456.

28. Donner-Banzhoff N, Echterhoff HH, Hense HW, Kunz R, Sawicki P, et al. (2000) Guidelines Clearing House statement "hypertension": summary and recommendations for a rational hypertension guideline in Germany [German]. Z Arztl Fortbild Qualitatssich 94: 341-349.

29. Eden J, Wheatley B, McNeil B, Sox H (2008) Selected findings from knowing what works in health care: a roadmap for the nation. Washington: Academic Press.

30. Faggion C Jr (2010) Grading the quality of evidence and the strength of recommendations in clinical dentistry: a critical review of 2 prominent approaches. J Evid Based Dent Pract 10: 78-85.

31. Feder G, Eccles M, Grol R, Griffiths C, Grimshaw J (1999) Clinical guidelines: using clinical guidelines. BMJ 318: 728-730.

32. Gerlach FM, Beyer M, Berndt M, Szecsenyi J, Abholz HH, et al. (1999) The DEGAM-concept: development, dissemination, implementation and evaluation of guidelines for general practice [German]. Z Arztl Fortbild Qualitatssich 93: 111-120.

33. Gillanders L, Angstmann K, Ball P, Chapman-Kiddell C, Hardy G, et al. (2008) AuSPEN clinical practice guideline for home parenteral nutrition patients in Australia and New Zealand. Nutrition 24: 998-1012.

34. Graham I, Harrison M, Brouwers M (2003) Evaluating and adapting practice guidelines for local use: a conceptual framework. In: Pickering S, Tomlinson J, editors. Clinical governance and best value: meeting the modernization agenda. London: Churchill Livingstone. pp. 213-229.

35. Graham ID, Harrison MB, Brouwers M, Davies BL, Dunn S (2002) Facilitating the use of evidence in practice: evaluating and adapting clinical practice guidelines for local use by health care organizations. J Obstet Gynecol Neonatal Nurs 31: 599-611.

36. Graham ID, Harrison MB, Lorimer K, Piercianowski T, Friedberg E, et al. (2005) Adapting national and international leg ulcer practice guidelines for local use: the Ontario Leg Ulcer Community Care Protocol. Adv Skin Wound Care 18: 307-318.

37. Green A, Kirk J (2007) Guidelines for the performance of the sweat test for the diagnosis of cystic fibrosis. Ann Clin Biochem 44: 25-34.

38. Green E, Zwaal C, Beals C, Fitzgerald B, Harle I, et al. (2010) Cancer-related pain management: a report of evidence-based recommendations to guide practice. Clin J Pain 26: 449-462.

39. Grimshaw J, Eccles M, Russell I (1995) Developing clinically valid practice guidelines. J Eval Clin Pract 1: 37-48.

40. Grol R, Cluzeau FA, Burgers JS (2003) Clinical practice guidelines: towards better quality guidelines and increased international collaboration. Br J Cancer 89(Suppl 1): S4-S8.

41. Groot P, Hommersom A, Lucas P (2008) Adaptation of clinical practice guidelines. Stud Health Technol Inform 139: 121-139.

42. Guo Y, Adelstein BA, Rubin GL (2007) Availability and development of guidelines in a tertiary teaching hospital. J Eval Clin Pract 13: 632-638.

43. Gupta S, Bhattacharyya OK, Brouwers MC, Estey EA, Harrison MB, et al. (2009) Canadian Thoracic Society: presenting a new process for clinical practice guideline production. Can Respir J 16: e62-e68.

44. Guyatt G, Oxman AD, Akl EA, Kunz R, Vist GE, et al. (2011) GRADE guidelines: 1; introduction; GRADE evidence profiles and summary of findings tables. J Clin Epidemiol 64: 383-394.

45. Guyatt GH, Oxman AD, Kunz R, Atkins D, Brozek J, et al. (2011) GRADE guidelines: 2; framing the question and deciding on important outcomes. J Clin Epidemiol 64: 395-400.

46. Guyatt GH, Oxman AD, Schünemann HJ, Tugwell P, Knottnerus A (2011) GRADE guidelines: a new series of articles in the Journal of Clinical Epidemiology. J Clin Epidemiol 64: 380-382.

47. Guyatt GH, Oxman AD, Vist GE, Kunz R, Brozek J, et al. (2011) GRADE guidelines: 4; rating the quality of evidence; study limitations (risk of bias). J Clin Epidemiol 64: 407-415.

48. Hanson D, Hoss BL, Wesorick B (2008) Evaluating the evidence: guidelines. AORN J 88: 184-196.

49. Harris JS, Sinnott PL, Holland JP, Ording J, Turkelson C, et al. (2008) Methodology to update the practice recommendations in the American College of Occupational and Environmental Medicine's Occupational Medicine Practice Guidelines, second edition. J Occup Environ Med 50: 282-295.

50. Harter M, Bermejo I, Ollenschläger G, Schneider F, Gaebel W, et al. (2006) Improving quality of care for depression: the German Action Programme for the implementation of evidence-based guidelines. Int J Qual Health Care 18: 113-119.

51. Helou A, Ollenschläger G (1998) Goals, possibilities and limits of quality evaluation of guidelines: a background report on the user manual of the "Methodological Quality of Guidelines" check list [German]. Z Arztl Fortbild Qualitatssich 92: 361-365.

52. Hermens RPMG, Ouwens MMTJ, Vonk-Okhuijsen SY, Van der Wel Y, Tjan-Heijnen VCG, et al. (2006) Development of quality indicators for diagnosis and treatment of patients with non-small cell lung cancer: a first step toward implementing a multidisciplinary, evidence-based guideline. Lung Cancer 54: 117-124.

53. Hill KM, Lalor EE (2009) How useful is an online tool to facilitate guideline implementation? Feasibility study of using eGLIA by stroke clinicians in Australia. Qual Saf Health Care 18: 157-159.

54. Horiuchi S, Yaju Y, Kataoka Y, Grace Eto H, Matsumoto N (2009) Development of an evidence-based domestic violence guideline: supporting perinatal women-centred care in Japan. Midwifery 25: 72-78.

55. Horvath AR, Nagy E, Watine J (2007) Critical appraisal of guidelines. In: Price C, Christenson R, editors. Evidence-based laboratory medicine: from principles to outcomes. Washington: AACC Press. pp. 295-320.

56. Hutchinson A, McIntosh A, Anderson J, Gilbert C, Field R (2003) Developing primary care review criteria from evidence-based guidelines: coronary heart disease as a model. Br J Gen Pract 53: 690-696.

57. Jarrett M (2009) Use of clinical practice guidelines to promote best practice when managing clinical interventions for liver transplant candidates. Prog Transplant 19: 132-140.

58. Jones R, Hunt C, Stevens R, Dalrymple J, Driscoll R, et al. (2009) Management of common gastrointestinal disorders: quality criteria based on patients' views and practice guidelines. Br J Gen Pract 59: 415-421.

59. Kiltz U, Feldtkeller E, Braun J (2008) Patient evaluation of the German version of the ASAS/EULAR recommendations for the management of ankylosing spondylitis [German]. Z Rheumatol 67: 694-700, 702.

60. Kirchner H, Fiene M, Ollenschläger G (2003) Assessment and implementation of guidelines [German]. Rehabilitation (Stuttg) 42: 74-82.

61. Kopp I (2008) Principles of the developmental process and implementation of guidelines: an update [German]. Radiologe 48: 1015-1016, 1018-1021.

62. Kopp IB (2010) Perspectives in guideline development and implementation in Germany [German]. Z Rheumatol 69: 298-304.

63. Krainovich-Miller B, Haber J, Yost J, Jacobs SK (2009) Evidence-based practice challenge: teaching critical appraisal of systematic reviews and clinical practice guidelines to graduate students. J Nurs Educ 48: 186-195.

64. Kryworuchko J, Stacey D, Bai N, Graham ID (2009) Twelve years of clinical practice guideline development, dissemination and evaluation in Canada (1994 to 2005). Implement Sci 4: 49.

65. Latoszek-Berendsen A, Tange H, Van den Herik HJ, Hasman A (2010) From clinical practice guidelines to computer-interpretable guidelines: a literature overview. Methods Inf Med 49: 550-570.

66. Lelgemann M, Thole H (2007) From evidence to recommendation: influence of consensus processes on grades of recommendation; 'against consensus'? [German]. Z Arztl Fortbild Qualitatssich 101: 97-101.

67. Littlejohns P, Cluzeau F (2000) Guidelines for evaluation. Fam Pract 17(Suppl 1): S3-S6.

68. MacDermid JC, Brooks D, Solway S, Switzer-McIntyre S, Brosseau L, et al. (2005) Reliability and validity of the AGREE instrument used by physical therapists in assessment of clinical practice guidelines. BMC Health Serv Res 5: 18.

69. Makela M (2006) Clinical guidelines: quality and implementation [German]. Z Arztl Fortbild Qualitatssich 100: 526-530.

70. Manchikanti L, Singh V, Derby R, Helm S, Trescot AM, et al. (2008) Review of occupational medicine practice guidelines for interventional pain management and potential implications. Pain Physician 11: 271-289.

71. McSweeney M, Spies M, Cann CJ (2001) Finding and evaluating clinical practice guidelines. Nurse Pract 26: 30, 33-34, 39, 43-44, 46-47.

72. Mendelson EB (1995) The development and meaning of appropriateness guidelines. Radiol Clin North Am 33: 1081-1084.

73. Mitglieder des Fachbereichs Pflege und Gesundheitsförderung des Deutschen Netzwerkes Evidenzbasierte Medizin (2006) Critical comment on nursing expert standards: meeting challenges to improve quality [German]. Pflege Z 59: 34-38.

74. Mourad SM, Hermens RPMG, Nelen WLDM, Braat DDM, Grol RPTM, et al. (2007) Guideline-based development of quality indicators for subfertility care. Hum Reprod 22: 2665-2672.

75. Muscarella LF (2007) Recommendations to resolve inconsistent guidelines for the reprocessing of sheathed and unsheathed rigid laryngoscopes. Infect Control Hosp Epidemiol 28: 504-507.

76. National Health and Medical Research Council (1999) A guide to the development, implementation and evaluation of clinical practice guidelines. Canberra: NHMRC. Available: http://www.nhmrc.gov.au/_files_nhmrc/publications/attachments/cp30.pdf.

77. Ollenschläger G (2000) Priority of health or treatment problems as themes of international guideline programs: proposition of the Guideline Clearing House on the evaluation of guidelines [German]. Z Arztl Fortbild Qualitatssich 94: 425-429.

78. Ollenschläger G, Kirchner H, Fiene M (2001) Practice guidelines in medicine: validity for clinical application? [German]. Internist 42: 473-474, 477-483.

79. Ollenschläger G, Thomeczek C, Bungart B, Lampert U, Arndt S, et al. (1999) The guideline clearing programme of the self-governmental bodies in the German health care system: a project to promote quality assurance in medicine [German]. Gesundheitswesen 61: 105-111.

80. Ollenschläger G, Thomeczek C, Kirchner H, Oesingmann U, Kolkmann FW, et al. (2001) The German Guidelines Clearing House (CGC): rationale, aims and results. Proc R Coll Phys Edinb 31(Suppl 9): 59-64.

81. Osonnaya C, Osonnaya K, Smith S, Swain P (2003) Developing guidelines for the management of helicobacter pylori infection in primary care: the consensus approach. International Journal of Medicine 5: 156-162.

82. Pagliaro U, Nieri M, Rotundo R, Cairo F, Carnevale G, et al. (2008) Clinical guidelines of the Italian Society of Periodontology for the reconstructive surgical treatment of angular bony defects in periodontal patients. J Periodontol 79: 2219-2232.

83. Pigni A, Brunelli C, Gibbins J, Hanks G, Deconno F, et al. (2010) Content development for European Guidelines on the use of opioids for cancer pain: a systematic review and Expert Consensus Study. Minerva Anestesiol 76: 833-843.

84. Poolman RW, Verheyen CCPM, Kerkhoffs GM, Bhandari M, Schünemann HJ (2009) From evidence to action: understanding clinical practice guidelines. Acta Orthop 80: 113-118.

85. Reed GM, McLaughlin CJ, Newman R (2002) American Psychological Association policy in context: the development and evaluation of guidelines for professional practice. Am Psychol 57: 1041-1047.

86. Rosenfeld RM, Shiffman RN (2006) Clinical practice guidelines: a manual for developing evidence-based guidelines to facilitate performance measurement and quality improvement. Otolaryngol Head Neck Surg 135(4 Suppl): S1-S28.

87. Rosser WW, Davis D, Gilbart E (2001) Assessing guidelines for use in family practice. J Fam Pract 50: 969-973.

88. Rossignol M, Poitras S, Dionne C, Tousignant M, Truchon M, et al. (2007) An interdisciplinary guideline development process: the Clinic on Low-back pain in Interdisciplinary Practice (CLIP) low-back pain guidelines. Implement Sci 2: 36.

89. Samama CM, Albaladejo P, Benhamou D, Bertin-Maghit M, Bruder N, et al. (2006) Venous thromboembolism prevention in surgery and obstetrics: clinical practice guidelines. Eur J Anaesthesiol 23: 95-116.

90. Serra A (2006) Critical analysis of upper respiratory tract infection guidelines. J Chemother 18: 60-61.

91. Shiffman R, Dixon J, Brandt C, Essaihi A, Hsiao A, et al. (2005) The GuideLine Implementability Appraisal (GLIA): development of an instrument to identify obstacles to guideline implementation. BMC Med Inform Decis Mak 5: 23.

92. Shinohara Y, Nagayama M, Origasa H (2009) Postpublication external review of the Japanese guidelines for the management of stroke 2004. Stroke 40: 1439-1443.

93. Simons MP, Aufenacker T, Bay-Nielsen M, Bouillot JL, Campanelli G, et al. (2009) European Hernia Society guidelines on the treatment of inguinal hernia in adult patients. Hernia 13: 343-403.

94. Singleton J, Levin R (2008) Strategies for learning evidence-based practice: critically appraising clinical practice guidelines. J Nurs Educ 47: 380-383.

95. Slavik R (2007) Clinical practice guidelines: critical appraisal is the key. Can J Hosp Pharm 60: 223-225,227-229.

96. Sprague AE, Oppenheimer L, McCabe L, Brownlee J, Graham ID, et al. (2006) The Ottawa Hospital's Clinical Practice Guideline for the Second Stage of Labour. J Obstet Gynaecol Can 28: 769-779.

97. Summers KH, Payakachat N (2006) Quality assessment of clinical practice guidelines. Expert Rev Pharmacoecon Outcomes Res 6: 1-4.

98. Thorson T, Mäkelä M (1999) Changing professional practice: theory and practice of clinical guidelines implementation. Copenhagen: Danish Institute for Health Services Research and Development.

99. Tremblay MS, Kho ME, Tricco AC, Duggan M (2010) Process description and evaluation of Canadian Physical Activity Guidelines development. International Journal of Behavioral Nutrition and Physical Activity 7: 42.

100. Tremblay MS, Leblanc AG, Janssen I, Kho ME, Hicks A, et al. (2011) Canadian sedentary behaviour guidelines for children and youth. Appl Physiol Nutr Metab 36: 59-64.

101. Tremblay MS, Warburton DE, Janssen I, Paterson DH, Latimer AE, et al. (2011) New Canadian physical activity guidelines. Appl Physiol Nutr Metab 36: 36-46.

102. Triano JJ (2008) Literature syntheses for the Council on Chiropractic Guidelines and Practice Parameters: methodology. J Manipulative Physiol Ther 31: 645-650.

103. Tugwell P, Pottie K, Welch V, Ueffing E, Chambers A, et al. (2010) Evaluation of evidence-based literature and formation of recommendations for the Clinical Preventive Guidelines for Immigrants and Refugees in Canada. CMAJ 183: E933-E938.

104. Vachhrajani S, Kulkarni AV, Kestle JRW (2009) Clinical practice guidelines. J Neurosurg Pediatrics 3: 249-256.

105. Van den Boogaard E, Goddijn M, Leschot NJ, Van der Veen F, Kremer JAM, et al. (2010) Development of guideline-based quality indicators for recurrent miscarriage. Reprod Biomed Online 20: 267-273.

106. Van der Sanden WJM, Mettes DG, Plasschaert AJM, Grol RPTM, Verdonschot EH (2004) Development of clinical practice guidelines: evaluation of 2 methods. J Can Dent Assoc 70: 301.

107. Voellinger R, Berney A, Baumann P, Annoni JM, Bryois C, et al. (2003) Major depressive disorder in the general hospital: adaptation of clinical practice guidelines. Gen Hosp Psychiatry 25: 185-193.

108. Von Troschke J, Selbmann HK, Encke A (2006) Health services research and guidelines: from the Association of the Scientific Medical Societies' (AWMF) perspective [German]. Z Arztl Fortbild Qualitatssich 100: 597-602.

109. Weinbrenner S, Lonnfors S, Babitsch B (2010) Gender: new methodological approaches in guideline development [German]. Z Evid Fortbild Qual Gesundhwes 104: 547-553.

110. Weingart O, Trapp H (2003) Evaluation of guidelines for treatment of breast cancer: guideline research system for the ÄZQ [German]. Onkologe 9: 1362-1365.

111. Woolf SH (1998) Do clinical practice guidelines define good medical care? The need for good science and the disclosure of uncertainty when defining 'best practices'. Chest 113(3 Suppl): 166S-171S.

112. Zhang W, Moskowitz RW, Nuki G, Abramson S, Altman RD, et al. (2007) OARSI recommendations for the management of hip and knee osteoarthritis, part I: critical appraisal of existing treatment guidelines and systematic review of current research evidence. Osteoarthritis Cartilage 15: 981-1000.

113. Zhang W, Moskowitz RW, Nuki G, Abramson S, Altman RD, et al. (2008) OARSI recommendations for the management of hip and knee osteoarthritis, part II: OARSI evidence-based, expert consensus guidelines. Osteoarthritis Cartilage 16: 137-162.

Comparison of guidelines without guideline appraisal

1. Ahn SS, Kim EK, Kang DR, Lim SK, Kwak JY, et al. (2010) Biopsy of thyroid nodules: comparison of three sets of guidelines. AJR Am J Roentgenol 194: 31-37.

2. Antonaci F, Dumitrache C, De Cillis I, Allena M (2010) A review of current European treatment guidelines for migraine. J Headache Pain 11: 13-19.

3. Beck C, Cody M, Souder E, Zhang M, Small GW (2000) Dementia diagnostic guidelines: methodologies, results, and implementation costs. J Am Geriatr Soc 48: 1195-1203.

4. Beghi E (2004) Efficacy and tolerability of the new antiepileptic drugs: comparison of two recent guidelines. Lancet Neurol 3: 618-621.

5. Blasi F, Ewig S, Torres A, Huchon G (2006) A review of guidelines for antibacterial use in acute exacerbations of chronic bronchitis. Pulm Pharmacol Ther 19: 361-369.

6. Boyd CM, Darer J, Boult C, Fried LP, Boult L, et al. (2005) Clinical practice guidelines and quality of care for older patients with multiple comorbid diseases: implications for pay for performance. JAMA 294: 716-724.

7. Burgers JS, Bailey JV, Klazinga NS, Van Der Bij AK, Grol R, et al. (2002) Inside guidelines: comparative analysis of recommendations and evidence in diabetes guidelines from 13 countries. Diabetes Care 25: 1933-1939.

8. Butchart EG (2009) Antithrombotic management in patients with prosthetic valves: a comparison of American and European guidelines. Heart 95: 430-436.

9. Cazzola M, Blasi F, Allegra L (2001) Critical evaluation of guidelines for the treatment of lower respiratory tract bacterial infections. Respir Med 95: 95-108.

10. Chauhan SP, Berghella V, Sanderson M, Magann EF, Morrison JC (2006) American College of Obstetricians and Gynecologists practice bulletins: an overview. Am J Obstet Gynecol 194: 1564-1572.

11. Chauhan SP, Gupta LM, Hendrix NW, Berghella V (2009) Intrauterine growth restriction: comparison of American College of Obstetricians and Gynecologists practice bulletin with other national guidelines. Am J Obstet Gynecol 200: 409.e1-409.e6.

12. Chiappini E, Regoli M, Bonsignori F, Sollai S, Parretti A, et al. (2011) Analysis of different recommendations from international guidelines for the management of acute pharyngitis in adults and children. Clin Ther 33: 48-58.

13. Cookson B, Mathai E, Allegranzi B, Pessoa-Silva CL, Bagheri Nejad S, et al. (2009) Comparison of national and subnational guidelines for hand hygiene. J Hosp Infect 72: 202-210.

14. Cutchie WA, Cheung NW, Simmons D (2006) Comparison of international and New Zealand guidelines for the care of pregnant women with diabetes. Diabet Med 23: 460-468.

15. De Gaudio M, Chiappini E, Galli L, De Martino M (2010) Therapeutic management of bacterial meningitis in children: a systematic review and comparison of published guidelines from a European perspective. J Chemother 22: 226-237.

16. DeVeber G (2005) In pursuit of evidence-based treatments for paediatric stroke: the UK and Chest guidelines. Lancet Neurol 4: 432-436.

17. Gensichen J, Huchzermeier C, Aldenhoff JB, Gerlach FM, Hinze-Selch D (2005) Signals for the initiation of structured diagnostic procedures for depression in primary health care: a practice-relevant evaluation of international guidelines [German]. Z Arztl Fortbild Qualitatssich 99: 57-63.

18. Georg G, Colombet I, Durieux P, Menard J, Meneton P (2008) A comparative analysis of four clinical guidelines for hypertension management. J Hum Hypertens 22: 829-837.

19. Grant J, Cottrell R, Cluzeau F, Fawcett G (2000) Evaluating "payback" on biomedical research from papers cited in clinical guidelines: applied bibliometric study. BMJ 320: 1107-1111.

20. Greer DM, Varelas PN, Haque S, Wijdicks EFM (2008) Variability of brain death determination guidelines in leading US neurologic institutions. Neurology 70: 284-289.

21. Gulsvik A, Gallefoss F, Dirksen A, Kinnula V, Gislason T, et al. (2006) A critical evaluation of the guidelines of obstructive lung disease and their implementation. Respir Med 100(Suppl 1): S22-S30.

22. Haagen EC, Hermens RPMG, Nelen WLDM, Braat DDM, Grol RPTM, et al. (2006) Subfertility guidelines in Europe: the quantity and quality of intrauterine insemination guidelines. Hum Reprod 21: 2103-2109.

23. Haertsch M, Campbell E, Sanson-Fisher R (1999) What is recommended for healthy women during pregnancy? A comparison of seven prenatal clinical practice guideline documents. Birth 26: 24-30.

24. Hanson L, VandeVusse L, Roberts J, Forristal A (2009) A critical appraisal of guidelines for antenatal care: components of care and priorities in prenatal education. J Midwifery Womens Health 54: 458-468.

25. Harvey RJ (1999) A review and commentary on a sample of 15 UK guidelines for the drug treatment of Alzheimer's disease. Int J Geriatr Psychiatry 14: 249-256.

26. Haverkate I, Muller MT, Cappetti M, Jonkers FJ, Van der Wal G (2000) Prevalence and content analysis of guidelines on handling requests for euthanasia or assisted suicide in Dutch nursing homes. Arch Intern Med 160: 317-322.

27. Iqbal A, Schloss S, George D, Isonaka S (2002) Worldwide guidelines for chronic obstructive pulmonary disease: a comparison of diagnosis and treatment recommendations. Respirology 7: 233-239.

28. Kaufer-Horwitz M, Valdes-Ramos R, Willett WC, Anderson A, Solomons NW (2005) A comparative analysis of the scientific basis and visual appeal of seven dietary guideline graphics. Nutr Res 25: 335-347.

29. Khan AR, Khan S, Zimmerman V, Baddour LM, Tleyjeh IM (2010) Quality and strength of evidence of the Infectious Diseases Society of America clinical practice guidelines. Clin Infect Dis 51: 1147-1156.

30. Koes BW, Van Tulder MW, Ostelo R, Kim Burton A, Waddell G (2001) Clinical guidelines for the management of low back pain in primary care: an international comparison. Spine 26: 2504-2513.

31. Lee DH, Vielemeyer O (2011) Analysis of overall level of evidence behind Infectious Diseases Society of America practice guidelines. Arch Intern Med 171: 18-22.

32. Manna DR, Bruijnzeels MA, Mokkink HGA, Berg M (2003) Ethnic specific recommendations in clinical practice guidelines: a first exploratory comparison between guidelines from the USA, Canada, the UK, and the Netherlands. Qual Saf Health Care 12: 353-358.

33. McAlister FA, Campbell NR, Zarnke K, Levine M, Graham ID (2001) The management of hypertension in Canada: a review of current guidelines, their shortcomings and implications for the future. CMAJ 164: 517-522.

34. McMurray J, Swedberg K (2006) Treatment of chronic heart failure: a comparison between the major guidelines. Eur Heart J 27: 1773-1777.

35. Moyer VA, Butler M (2004) Gaps in the evidence for well-child care: a challenge to our profession. Pediatrics 114: 1511-1521.

36. Müller U, Wolf H, Kiefer M, Gertz HJ (2003) A systematic comparison of national and international dementia guidelines [German]. Fortschr Neurol Psychiatr 71: 285-295.

37. Munoz M, Estevez LG, Alvarez I, Fernandez Y, Margeli M, et al. (2008) Evaluation of international treatment guidelines and prognostic tests for the treatment of early breast cancer. Cancer Treat Rev 34: 701-709.

38. Myers TR (2008) Guidelines for asthma management: a review and comparison of 5 current guidelines. Respir Care 53: 751-767.

39. O'Brien E, Staessen JA (2000) Critical appraisal of the JNC VI, WHO/ISH and BHS guidelines for essential hypertension. Expert Opin Pharmacother 1: 675-682.

40. Oliveira AG (2005) Current management of hospitalized community acquired pneumonia in Portugal: consensus statements of an expert panel. Rev Port Pneumol 11: 243-282.

41. Parkes R, Renton A, Meheus A, Laukamm-Josten U (2004) Review of current evidence and comparison of guidelines for effective syphilis treatment in Europe. Int J STD AIDS 15: 73-88.

42. Perlis RH (2005) The role of pharmacologic treatment guidelines for bipolar disorder. J Clin Psychiatry 66(Suppl 3): 37-47.

43. Persons JB, Thase ME, Crits-Christoph P (1996) The role of psychotherapy in the treatment of depression: review of two practice guidelines. Arch Gen Psychiatry 53: 283-290.

44. Pignotti MS, Donzelli G (2008) Perinatal care at the threshold of viability: an international comparison of practical guidelines for the treatment of extremely preterm births. Pediatrics 121: e193-e198.

45. Rossignol M, Moride Y, Perreault S, Boivin JF, Ste-Marie LG, et al. (2002) Recommendations for the prevention of osteoporosis and fragility fractures: international comparison and synthesis. Int J Technol Assess Health Care 18: 597-610.

46. Santamaria R, Martinez E, Kratochwill S, Soria C, Tan LH, et al. (2009) Comparison and critical appraisal of dengue clinical guidelines and their use in Asia and Latin America. International Health 1: 133-140.

47. Scholes D, Anderson LA, Operskalski BH, BlueSpruce J, Irwin K, et al. (2003) STD prevention and treatment guidelines: a review from a managed care perspective. Am J Manag Care 9: 181-189.

48. Schuurmans A, Van Weel C (2005) Pharmacologic treatment of migraine: comparison of guidelines. Can Fam Physician 51: 838-843.

49. Silva AL, Marinho MR, Gouveia FM, Silva JG, Ferreira Ade S, et al. (2011) Benign Paroxysmal Positional Vertigo: comparison of two recent international guidelines. Braz J Otorhinolaryngol 77: 191-200.

50. Silvester JA, Rashid M (2007) Long-term follow-up of individuals with celiac disease: an evaluation of current practice guidelines. Can J Gastroenterol 21: 557-564.

51. Solomon DH, Morris C, Cheng H, Cabral D, Katz JN, et al. (2005) Medication use patterns for osteoporosis: an assessment of guidelines, treatment rates, and quality improvement interventions. Mayo Clin Proc 80: 194-202.

52. Stern JM (2009) Overview of evaluation and treatment guidelines for epilepsy. Curr Treat Options Neurol 11: 273-284.

53. Thomas D, Giugliano RP (2009) Management of ST-segment elevation myocardial infarction: comparison of the updated guidelines from North America and Europe. Am Heart J 158: 695-705.

54. Thomsen HS, Morcos SK (2006) Contrast-medium-induced nephropathy: is there a new consensus? A review of published guidelines. Eur Radiol 16: 1835-1840.

55. Van Hecke A, Grypdonck M, Defloor T (2008) Guidelines for the management of venous leg ulcers: a gap analysis. J Eval Clin Pract 14: 812-822.

56. Van Wyk JT, Van Wijk MAM (2004) Analysis of Dutch general practice guidelines for inconsistencies with respect to the management of cardiovascular disease risk factors. Stud Health Technol Inform 107: 179-186.

57. Vanbelleghem H, Vanholder R, Levin NW, Becker G, Craig JC, et al. (2007) The Kidney Disease: improving Global Outcomes website; comparison of guidelines as a tool for harmonization. Kidney Int 71: 1054-1061.

58. Vogel N, Burnand B, Vial Y, Ruiz J, Paccaud F, et al. (2000) Screening for gestational diabetes: variation in guidelines. Eur J Obstet Gynecol Reprod Biol 91: 29-36.

59. Wennekes L, Hermens RPMG, Van Heumen K, Runde V, Schoelen H, et al. (2008) Possibilities for transborder cooperation in breast cancer care in Europe: a comparative analysis regarding the content, quality and evidence use of breast cancer guidelines. Breast 17: 464-471.

60. Winn RJ, Brown NH, Botnick WZ (1999) Reproducibility of guidelines: a comparison of the NCCN and ASCO lung cancer guidelines. Oncology (Williston Park) 13: 35-39.

61. Young T (2000) Critical appraisal of pressure ulcer guidelines. Community Nurse 5: 29-30.

Application of guideline recommendations without guideline appraisal

1. Asukata Y, Ishihara M, Hasumi Y, Nakamura S, Hayashi K, et al. (2008) Guidelines for the diagnosis of ocular sarcoidosis. Ocul Immunol Inflamm 16: 77-81.

2. Bosson JL, Labarere J (2006) Determining indications for care common to competing guidelines by using classification tree analysis: application to the prevention of venous thromboembolism in medical inpatients. Med Decis Making 26: 63-75.

3. Broedl UC, Geiss HC, Parhofer KG (2003) Comparison of current guidelines for primary prevention of coronary heart disease: risk assessment and lipid-lowering therapy. J Gen Intern Med 18: 190-195.

4. Friedman SE, Palac RT, Zlotnick DM, Chobanian MC, Costa SP (2011) A call to action: variability in guidelines for cardiac evaluation before renal transplantation. Clin J Am Soc Nephrol 6: 1185-1191.

5. Gillois P, Claudot F, Chatellier G, Kohler F, Jaulent MC (2006) Comparison of the impact of cardiovascular guidelines on a working population. Stud Health Technol Inform 124: 639-644.

6. Ioannides-Demos LL (1998) Drug use evaluation of vancomycin based on hospital policy and published guidelines. Australian Journal of Hospital Pharmacy 28: 394-399.

7. Julka PK, Doval DC, Gupta S, Rath GK (2008) Response assessment in solid tumours: a comparison of WHO, SWOG and RECIST guidelines. Br J Radiol 81: 444-449.

8. Kelly AM, Druda D (2008) Comparison of size classification of primary spontaneous pneumothorax by three international guidelines: a case for international consensus? Respir Med 102: 1830-1832.

9. Kyoong A, Mol S, Guy P, Finlay P, Strauss BJ, et al. (2006) Comparison of Australian and international guidelines for grading severity of chronic obstructive pulmonary disease. Intern Med J 36: 506-512.

10. Labarere J, Bosson JL, Bergmann JF, Thilly N (2004) Agreement of four competing guidelines on prevention of venous thromboembolism and comparison with observed physician practices: a cross-sectional study of 1,032 medical inpatients. J Gen Intern Med 19: 849-855.

11. Lin GA, Redberg RF, Anderson HV, Shaw RE, Milford-Beland S, et al. (2010) Impact of changes in clinical practice guidelines on assessment of quality of care. Med Care 48: 733-738.

12. Manuel DG, Kwong K, Tanuseputro P, Lim J, Mustard CA, et al. (2006) Effectiveness and efficiency of different guidelines on statin treatment for preventing deaths from coronary heart disease: modelling study. BMJ 332: 1419.

13. Sheehy AM, Flood GE, Tuan WJ, Liou J, Coursin DB, et al. (2010) Analysis of guidelines for screening diabetes mellitus in an ambulatory population. Mayo Clin Proc 85: 27-35.

14. Skrifvars MB, Vayrynen T, Kuisma M, Castren M, Parr MJ, et al. (2010) Comparison of Helsinki and European Resuscitation Council "do not attempt to resuscitate" guidelines, and a termination of resuscitation clinical prediction rule for out-of-hospital cardiac arrest patients found in asystole or pulseless electrical activity. Resuscitation 81: 679-684.

15. Subramanian A, Thomasson L, Hanson H, Hodgson S, Simson JNL (2008) The BSG/ACPGBI guidelines for colonoscopic screening: what are we missing? Colorectal Dis 10: 673-676.

16. Thomson R, McElroy H, Sudlow M (1998) Guidelines on anticoagulant treatment in atrial fibrillation in Great Britain: variation in content and implications for treatment. BMJ 316: 509-513.

17. Von Eckardstein A, Schulte H, Assmann G (2005) Comparison of international recommendations for the recognition of asymptomatic high risk patients for a heart attack in Germany [German]. Z Kardiol 94: 52-60.

18. Yoon J, Kwon SR, Lim MJ, Joo K, Moon CG, et al. (2010) A comparison of three different guidelines for osteoporosis treatment in patients with rheumatoid arthritis in Korea. Korean J Intern Med 25: 436-446.

19. Yu HR, Niu CK, Kuo HC, Tsui KY, Wu CC, et al. (2010) Comparison of the Global Initiative for Asthma guideline-based asthma control measure and the Childhood Asthma Control Test in evaluating asthma control in children. Pediatr Neonatol 51: 273-278.
