# Supplementary material for: Appraisal Tools for Clinical Practice Guidelines: A Systematic Review
Source: PLoS One. 2013 Dec 9;8(12):e82915. doi: 10.1371/journal.pone.0082915 (PMC3857289; doi:10.1371/journal.pone.0082915)
Supplement: File S3 — Relevant secondary publications. (PDF) [file pone.0082915.s004.pdf]

Supporting information 3 – Relevant secondary publications

1. Graham ID, Calder LA, Hebert PC, Carter AO, Tetroe JM (2000) A comparison of clinical practice guideline appraisal instruments. Int J Technol Assess Health Care 16: 1024-1038.

2. Vlayen J, Aertgeerts B, Hannes K, Sermeus W, Ramaekers D (2005) A systematic review of appraisal tools for clinical practice guidelines: multiple similarities and one common deficit. Int J Qual Health Care 17: 235-242.
